# Tissue-specific and interpretable sub-segmentation of whole tumour burden on CT images by unsupervised fuzzy clustering

**DOI:** 10.1016/j.compbiomed.2020.103751

**Published:** 2020-05

**Authors:** Leonardo Rundo, Lucian Beer, Stephan Ursprung, Paula Martin-Gonzalez, Florian Markowetz, James D. Brenton, Mireia Crispin-Ortuzar, Evis Sala, Ramona Woitek

**Affiliations:** a Department of Radiology, University of Cambridge, Cambridge CB2 0QQ, UK; b Cancer Research UK Cambridge Centre, University of Cambridge, Cambridge CB2 0RE, UK; c Cancer Research UK Cambridge Institute, University of Cambridge, Cambridge CB2 0RE, UK; d Department of Biomedical Imaging and Image-guided Therapy, Medical University Vienna, Vienna 1090, Austria

**Keywords:** Tissue-specific segmentation, Computed tomography, Unsupervised fuzzy clustering, Ovarian cancer, Renal cell carcinoma, Radiomics

## Abstract

**Background:**

Cancer typically exhibits genotypic and phenotypic heterogeneity, which can have prognostic significance and influence therapy response. Computed Tomography (CT)-based radiomic approaches calculate quantitative features of tumour heterogeneity at a mesoscopic level, regardless of macroscopic areas of hypo-dense (i.e., cystic/necrotic), hyper-dense (i.e., calcified), or intermediately dense (i.e., soft tissue) portions.

**Method:**

With the goal of achieving the automated sub-segmentation of these three tissue types, we present here a two-stage computational framework based on unsupervised Fuzzy C-Means Clustering (FCM) techniques. No existing approach has specifically addressed this task so far. Our tissue-specific image sub-segmentation was tested on ovarian cancer (pelvic/ovarian and omental disease) and renal cell carcinoma CT datasets using both overlap-based and distance-based metrics for evaluation.

**Results:**

On all tested sub-segmentation tasks, our two-stage segmentation approach outperformed conventional segmentation techniques: fixed multi-thresholding, the Otsu method, and automatic cluster number selection heuristics for the *K*-means clustering algorithm. In addition, experiments showed that the integration of the spatial information into the FCM algorithm generally achieves more accurate segmentation results, whilst the kernelised FCM versions are not beneficial. The best spatial FCM configuration achieved average Dice similarity coefficient values starting from 81.94±4.76 and 83.43±3.81 for hyper-dense and hypo-dense components, respectively, for the investigated sub-segmentation tasks.

**Conclusions:**

The proposed intelligent framework could be readily integrated into clinical research environments and provides robust tools for future radiomic biomarker validation.

## Highlights

• Intelligent two-stage method for CT tissue-specific image segmentation of whole tumours.
• Unsupervised fuzzy clustering framework for interpretable sub-segmentation.
• Sub-segmentation results achieved on ovarian and renal cancer are accurate and reliable.
• The proposed computational framework considerably outperforms conventional approaches.
• Insights gained about intra-tumoural heterogeneity evaluation for radiomics.

## Graphical abstract

**Figure:**
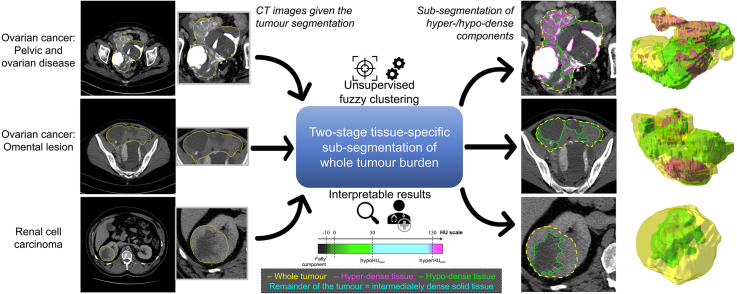

## 1 Introduction

**Fig. 1:**
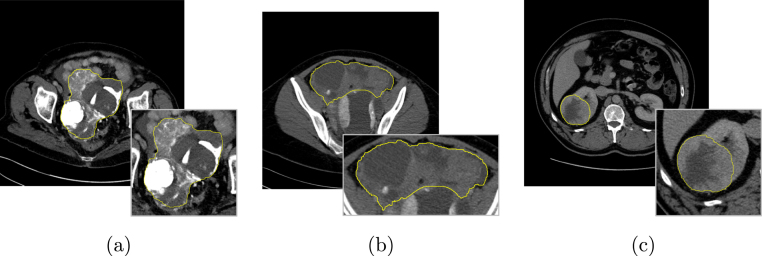
Examples of input axial CT slices for tissue-specific sub-segmentation: (a, b) HGSOC lesions in the pelvis and omentum, respectively; (c) RCC. The whole tumour burden, defined by the yellow contour and zoomed at the bottom right of each sub-figure, is characterised by mixed tumoural tissues.

Cancer is typically characterised by genotypic and phenotypic heterogeneity, which has prognostic significance and may influence the response to therapy [1]. Computed Tomography (CT) is the most frequently used cross-sectional imaging method in oncology. It quantifies spatial variation in the morphology of individual tumours by measuring variations in X-ray attenuation, which allows for the assessment of the macro- and mesoscopic structure of tumours [2], [3]. Intra- and inter-tumoural heterogeneity can be quantified on the mesoscopic level by using CT-based radiomics, which has been shown to hold both predictive and prognostic information for many cancer types, including high-grade serous ovarian carcinoma (HGSOC) and renal cell carcinoma (RCC). Notably, these two cancer types are characterised by high levels of macroscopic heterogeneity with frequent cystic/necrotic, solid, and calcified tumour regions [3], [4], [5], [6], [7], [8], [9]. However, the majority of radiomics studies disregard macroscopic tumour heterogeneity, even though solid tumour regions typically have high cellular density and could contribute more to adverse prognostic or predictive information than necrotic, cystic, or calcified regions [10]. We reasoned that applying different weightings to radiomic features for macroscopically different tumour regions could increase accuracy for predicting response and outcome. However, clinical CT reporting to evaluate the size of tumour masses and response to treatment relies upon mono- or multi-dimensional tumour diameters, typically following RECIST 1.1 criteria [11]. This standard reporting does not quantify the proportion of the tumour that is composed of solid, cystic/necrotic, or calcified tissue [11]. These methods therefore may benefit from automated or manual sub-segmentation of all disease present. Fig. 1 shows three examples of axial CT slices analysed for tissue-specific sub-segmentation of HGSOC and RCC lesions.

Recent advances in machine learning techniques for medical imaging have benefited from the strong learning ability of fully supervised deep learning models [12] and the availability of large training datasets that include accurate and detailed annotations [13], [14]. In order to work on datasets with less accurate annotations (for example, bounding boxes or image-level labels [15]), different machine learning models use weak supervision [16] or Generative Adversarial Networks (GANs) [17], [18] for data augmentation. In clinical applications, particularly in the case of heterogeneous or multi-institutional datasets, the development of effective supervised deep models typically relies upon solutions tailored for obtaining adequate generalisation abilities, even on limited data samples [19], [20]. For this reason, when dealing with an amount of labelled input data that does not allow for a representative training sample along with a sufficient unseen test set, unsupervised machine learning techniques have particularly gained ground in biomedical applications [21], [22].

We mainly address the following issue in medical image analysis:

• How accurately does an unsupervised machine learning approach segment hyper-dense and hypo-dense components on the whole tumour burden on CT images?

The rationale underlying this question, towards precision oncology, was:

• May tissue-specific cancer sub-segmentation, as a measure of intra-tumoural heterogeneity, provide insights into a more precise therapy response assessment?

In this work, we propose a computational framework based on unsupervised machine learning techniques to sub-segment tumour lesions into hypo-dense (cystic/necrotic), hyper-dense (calcified), and intermediately dense (soft tissue) tumour components. To the best of our knowledge, this is the first approach that purposely focuses on whole tumour burden sub-segmentation on CT images. Our method optimises the segmentation for each individual image whilst also taking into account prior domain knowledge for the typical densities of candidate sub-regions. Our automated approach allows for deployment in clinical research environments, without the need for any training phase [23]. Furthermore, the results of our tissue-specific sub-segmentation method are interpretable by researchers and clinicians [24], [25], by taking into account prior domain knowledge of the typical sub-region Hounsfield Unit (HU) values.

This manuscript is organised as follows. Section 2 concisely introduces the theoretical background of unsupervised fuzzy clustering techniques. The proposed automatic tissue-specific segmentation framework is presented in Section 3. Section 4 presents the characteristics of the analysed HGSOC and RCC datasets, along with the evaluation methodology used. Section 5 shows and discusses the achieved experimental results. Finally, Section 6 provides conclusive remarks and future directions.

## 2 Unsupervised fuzzy clustering techniques

This section briefly outlines the main concepts underlying the devised unsupervised fuzzy clustering framework designed to unify the classic, spatial, and kernelised versions of the Fuzzy C-Means (FCM) method [26], [27]. For a detailed description of the mathematical formulation, please refer to Section S1 in the Supplementary Material.

Fundamentally, the FCM algorithm [26], [27] is a partitional clustering technique that minimises the intra-cluster variance, as well as maximises the inter-cluster variance, in terms of a distance metric between the feature vectors [28]. This unsupervised technique optimises the intrinsic partitioning of an unlabelled dataset $X=\left\{x_{1},x_{2},\ldots ,x_{N}\right\}$ composed of N feature vectors, which denote data samples $x_{k}\in R^{D}$ (k=1,2,…,N) belonging to a D-dimensional Euclidean space, into exactly C clusters (i.e., non-empty partitions of the input dataset). Thus, a partition P is defined as a fuzzy set family $P=\left\{Y_{1},Y_{2},\ldots ,Y_{C}\right\}$ [29]. Importantly, let $V=\left\{v_{1},v_{2},\ldots ,v_{C}\right\}$ be a set of D-dimensional prototype vectors, called centroids that are associated with the C clusters. Therefore, the input dataset X is partitioned by iteratively searching for the optimal fuzzy partition P that minimises an objective function $J_{m}$ (where m denotes the fuzzification constant) by means of a local optimisation technique. The role of the weighting exponent m in the FCM model was systematically analysed in [30], where the authors suggested that the best choice for m is in the interval [1.5,2.5], and its mean value m=2 is typically used.

The classic FCM clustering algorithm does not take into account any spatial relationship among pixels since all the samples are used as disperse and independent points, making it sensitive to noise and other imaging artefacts [31]. Accordingly, the integration of spatial information might be beneficial. The spatial FCM (sFCM), elegantly introduced by Chuang et al. [32], enables the retention of the same formulation and objective function as the classic FCM algorithm, just by modifying the update rules with the local spatial content in the image. The incorporation of this spatial component considerably improves the performance: (*i*) in a homogeneous region, the spatial functions emphasise the original membership, so the clustering results are not affected; (*ii*) in noisy regions, spurious blobs or misclassified pixels may be corrected. According to [32], in all the tests, a local squared window of ω×ω pixels, with ω=5, was used. Simply, the parameters p and q weight the original membership (based on pixel values alone) and spatial components, respectively. Hereafter, in compliance with the notation introduced in [32], we denote the sFCM with the control parameters p and q as sFCM${}_{p,q}$. Relying upon the literature [31], [32], we tested p=1 and q∈{0,1,2}.

**Fig. 2:**
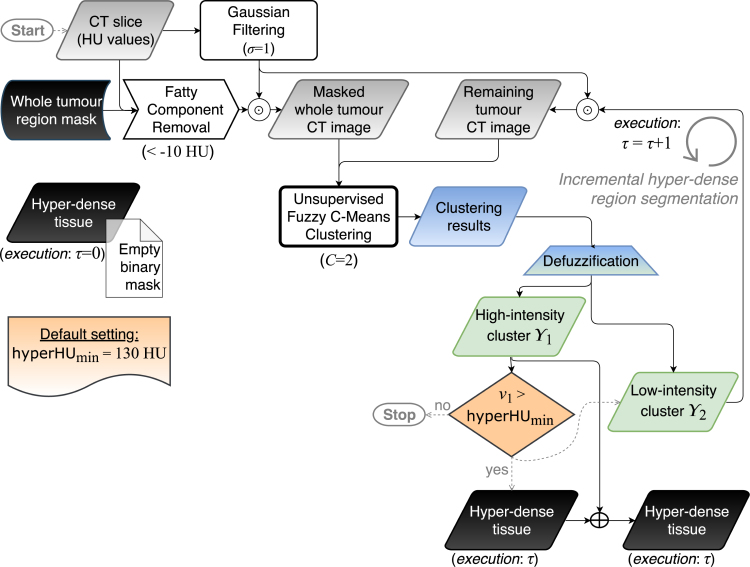
Flow diagram of the hyper-dense tissue segmentation (i.e., phase 1). The grey and black data blocks denote grey-scale images and binary masks, respectively. The gradient blue–green trapezoidal block represents the defuzzification step, *via* a maximum membership scheme, from the fuzzy clustering results (blue data block) to the crisp clusters (green data blocks). Solid and dashed arrows correspond to processing and control-oriented operations, respectively.

The metric used in the objective function of these FCM versions is still the Euclidean distance. However, the use of the $\ell_{2}$ norm might lead to non-robust results on the segmentation of an image corrupted by noise, outliers, and other imaging artefacts. The kernelised methods let us generalise distance-based algorithms to operate in feature spaces, usually non-linearly related to the input space. Importantly, kernelised methods are suitable for clustering algorithms [33] and also allow for implicit mapping [34]. In our implementation, a Gaussian Radial Basis Function (GRBF) kernel was used:

(1) $$K\left(x,y\right)=e^{-\frac{{\left\|x-y\right\|}^{2}}{2\sigma^{2}}},$$

where σ denotes the kernel width. Since σ is a particularly sensitive parameter we relied upon [33], where an adaptive strategy is used to determine the kernel parameters by using the fast bandwidth selection rule in Eq. (2), based on the distance variance of all data points in the collection:

(2) $$\sigma =\sqrt{\frac{1}{N-1}\overset{N}{\underset{i=1}{\sum }}{\left(d_{i}-\overset{¯}{d}\right)}^{2}},$$

where $d_{i}=\|x_{i}-\overset{¯}{x}\|$ is the distance from the grey-scale of the ith pixel to the grey-scale average of all pixels and $\overset{¯}{d}$ is the average of all distances $d_{i}$. To perform a comparison independent of centroid initialisation, our kernelised sFCM (ksFCM) version exploited the formulation adopted by the classic FCM algorithm in the original space.

For all the implemented fuzzy clustering methods, the convergence conditions can be defined by comparing the value of the objective function $J_{m}$ between two consecutive iterations. The iterative procedure ends when the convergence condition is less than a fixed tolerance value $\varepsilon_{\text{tol}}$ or the maximum number of allowed iterations $T_{\text{max}}$ is achieved. In all the tests, we used $\varepsilon_{\text{tol}}=10^{-5}$ and $T_{\text{max}}=100$.

Regarding the computational complexity (for each iteration), the classic FCM algorithm requires O(NCD) floating-point operations [35], [36]. With the introduction of the spatial information conveyed by the local ω×ω window, the sFCM version has a time complexity of $O\left(NCD+N\omega^{2}\right)$. The ksFCM version involves also the kernel distance computation characterised by a quadratic complexity with the number of objects N, resulting in $O\left(N^{2}CD+N\omega^{2}\right)$ floating-point operations [36], [37].

In the literature, additional solutions have been proposed to deal with large datasets. Cannon et al. in [38] proposed the approximate FCM to reduce the FCM’s time complexity by replacing the exact calculation with approximate ones *via* look-up tables for the Euclidean distances and exponentiation operations. However, these approximations can be relevant mostly for integer-valued data, whilst lead to result quality degradation for real-valued data [35]. In terms of memory reduction, the reformulation of the iterative FCM update steps presented in [35] allows for eliminating the storage of the membership matrix $U\in R^{C\times N}$. Nevertheless, our implementation stores this data structure for the membership filtering that considers the spatial neighbourhood for each pixel.

Recent FCM-based techniques mostly aim at improving the search and convergence capabilities of the optimisation process. Careful seeding mechanisms, such as the FCM＋＋ approach [39], adaptively scatter the initial cluster centroids throughout the data space during the initialisation phase. To further boost the FCM performance, extensions and modifications to the objective function can be introduced. For instance, hyper-volume prototypes (with size either fixed or determined automatically from the data undergoing clustering) and heuristic-based adaptive cluster merging or incremental fuzzy partitioning were introduced in [40] and [41], respectively. Alternatively, the search for the optimal solution could be improved by replacing gradient-based search techniques with global optimisation techniques, such as evolutionary strategies [42] or Particle Swarm Optimisation (PSO) [43]. However, these metaheuristics (i.e., population-based stochastic optimisation techniques) are strongly affected by the initialisation of the solutions’ population, by influencing both the convergence speed and the quality of the solutions [44], [45], as well as careful functioning parameter settings [46]. In this direction, Mekhmoukh and Mokrani in [47] exploited the PSO algorithm for the initial choice of the cluster centroids in brain tissue segmentation on Magnetic Resonance Imaging (MRI) scans. Finally, the fuzzy clustering result was refined *via* level set functions.

**Fig. 3:**
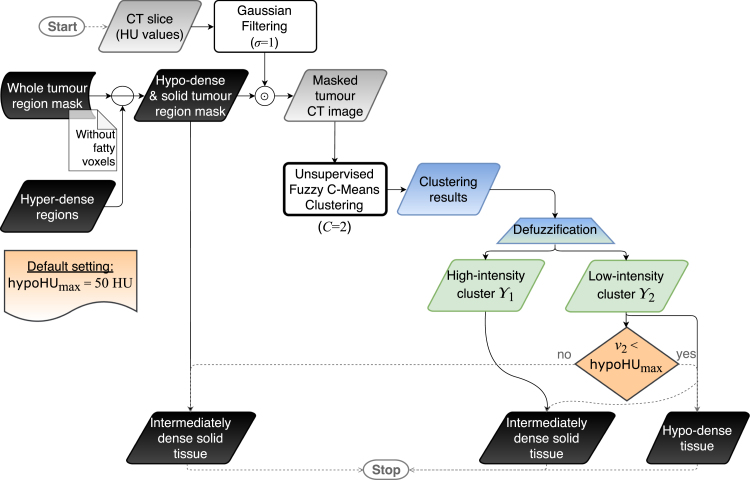
Flow diagram of the hypo-dense tissue segmentation (i.e., phase 2). The gradient blue–green trapezoidal block represents the defuzzification step, *via* a maximum membership scheme, from the fuzzy clustering results (blue data block) to the crisp clusters (green data blocks). Solid and dashed arrows correspond to processing and control-oriented operations, respectively.

In our experiments, the initial fuzzy partitions were randomly generated to carry out a fair comparison independent of centroid initialisation, thus ensuring result repeatability among the unsupervised fuzzy clustering versions investigated in the proposed framework. Moreover, no further computational burden was introduced by careful initialisation schemes.

## 3 The proposed tissue-specific CT image segmentation method

In our tissue-specific CT image segmentation method, we decided to consider the HU values alone for the segmentation – without including any texture feature (e.g., Haralick features [48], [49]) – in order to obtain interpretable results and avoid possible biases in the downstream radiomics analysis (particularly, for feature selection in biomarker development). In this manner, this design choice decouples the morphological tissue-specific sub-segmentation from radiomics-based habitat analyses, as well as maintains the interpretability of the cluster centroids expressed in HU (which are fully understandable for the end-user). Therefore, from now on, the cluster centroids are denoted as scalars $v_{i}\in V\subseteq R^{C}$.

As a simple pre-processing step, a Gaussian filter (with σ=1) was applied by means of a 5 × 5 convolution kernel. In order to deal with the high bias in the hypo-dense and hyper-dense tissue detection based on unsupervised clustering, a pre-processing step to remove the fatty components was performed; more specifically, the voxels with values lower than −10 HU are removed. This strategy deals with the possible errors in the delineation process (mainly due to the discretisation of the contour drawn by the radiologists that outlines tumours that are surrounded by non-cancerous fat tissue).

The overall sub-segmentation method, relying upon previously delineated whole tumour region masks, leverages a *divide-et-impera* strategy *via* two stages represented in Fig. 2, Fig. 3, respectively:

1. Detection of the hyper-dense regions: multiple executions τ of the unsupervised clustering with C=2, by incrementally including clusters in which the centroid $v_{1}$ is higher than the minimum hyper-dense cluster selection value ${\mathrm{hyperHU}}_{\text{min}}$. This iterative procedure takes into account the heterogeneity of the hyper-dense tissues;
2. Distinction between hypo-dense and intermediately dense regions: the clustering algorithm is executed with C=2. Afterwards, the minimum intensity centroid $v_{2}$ is compared with the maximum hypo-dense cluster selection value ${\mathrm{hypoHU}}_{\text{max}}$.

This two-stage approach allows us to efficiently avoid the estimation of the number of clusters *via* heuristics, since C is unknown *a priori*. The sequential order of the two phases is motivated by detection purposes of hyper-dense components, which might present small/diffuse calcifications. Besides, the larger HU range of hyper-dense tissues with respect to hypo-dense portions (even hundreds *versus* few tens in terms of HU value ranges) justifies the choice of multiple executions of the clustering procedure (during phase 1), particularly in the case of highly calcified sub-regions. Afterwards, the delineation of hypo-dense regions can be performed suitably.

**Fig. 4:**
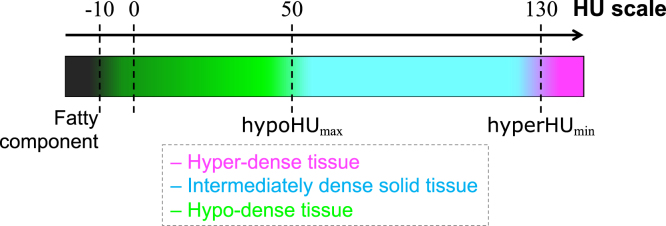
HU scale showing the different densities of the tissues composing the whole tumour burden on CT imaging. ${\mathrm{hyperHU}}_{\text{min}}$ and ${\mathrm{hypoHU}}_{\text{max}}$ denote the cluster selection values for the hyper-dense and hypo-dense tissues, respectively. The colour legend is shown at the bottom.

To determine the best settings for the cluster selection values, we considered a calibration set consisting of HGSOC lesions containing both hyper-dense and hypo-dense regions. Only two RCC lesions included small calcified areas (see Section 4.1.2) and we used this dataset as an external validation. As a baseline, we used the classic FCM algorithm (i.e., sFCM${}_{1,0}$) without any morphological post-processing to focus on the performance depending only on cluster selection values. The value of ${\mathrm{hyperHU}}_{\text{min}}$ varied in {110,120,130,140,150} HU considering a calibration set of 70 randomly selected CT images with hyper-dense components. After selecting the best ${\mathrm{hyperHU}}_{\text{min}}$, the ${\mathrm{hypoHU}}_{\text{max}}$ values in {20,30,40,50,60} HU were tested on a calibration set composed of 120 randomly selected CT images with hypo-dense components (since hypo-dense tissue is more frequent than hyper-dense regions, as described in Section 4.1). In this study, relying upon the results in supplementary Figs. S1 and S2, the cluster selection values ${\mathrm{hyperHU}}_{\text{min}}$ and ${\mathrm{hypoHU}}_{\text{max}}$ were set to 130 HU and 50 HU, respectively, to achieve the best compromise in terms of correct detection performance and reliability, *via* the Dice similarity coefficient (DSC) explained in Section 4.2.2. In more detail, the trend of ${\mathrm{hyperHU}}_{\text{min}}$ shows a degradation of DSC values for 140 and 150 HU since small calcifications might be overlooked. In the case of ${\mathrm{hypoHU}}_{\text{max}}$, a value of 20 HU misses the majority of the hypo-dense components, whilst 50 HU shows the lowest standard deviation. Accordingly, we aimed to show the robustness of these settings on unseen data, especially in the case of the RCC dataset. Fig. 4 illustrates the interpretability of our approach *via* a colour-coded HU scale of the different tissues composing the whole tumour burden. Gradient colours were used to show that no fixed threshold can reliably identify the hyper-dense and hypo-dense components. A maximum membership defuzzification scheme was applied after every unsupervised fuzzy clustering execution to yield a crisp classification.

This two-stage approach ensures robustness in highly variable clinical scenarios, such as in the case of metastatic HGSOC that is frequently composed of up to three different tissue types. Using this *divide-et-impera* strategy, no technique for automatic selection of the number of clusters is needed. In fact, the inherent variability within the analysed cohort of patients and tumour types, considering both the different acquisition parameters and tissues occurring in the lesions, might affect the reliability in the estimation of the number of clusters. These strategies include heuristics (e.g., elbow or silhouette methods), information theory methods, or fuzzy clustering validity measures [28], [50].

The proposed two-stage approach was applied separately to each lesion type (even when there was more than one distinct lesion in a given CT slice). Furthermore, to increase accuracy, the unsupervised fuzzy clustering was performed independently on each connected-component of the whole tumour. This is important when the regions split or merge across adjacent slices, which is particularly the case in HGSOC.

The proposed approach was developed using the MatLab® R2019b (64-bit version) environment (The MathWorks, Natick, MA, USA). The tests were conducted on a MacOS X (Mojave, version 10.14.6) computational platform equipped with an Intel® Core™ i7@2.7 GHz CPU and 16 GB of RAM.

### 3.1 Hyper-dense tissue segmentation

**Fig. 5:**
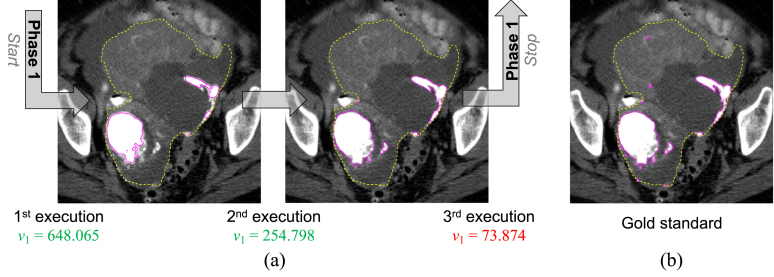
Incremental hyper-dense region segmentation *via* multiple executions of the unsupervised fuzzy clustering models for hyper-dense region detection. (a) Example of intermediate results obtained by the first phase of the proposed pipeline (employing sFCM${}_{1,1}$) on an HGSOC CT image. The high-intensity cluster centroid values $v_{1}$, during the executions, are also shown. For better clarity, the green-coloured and red-coloured centroids $v_{1}$ denote higher or lower values than the selected cluster selection value ${\mathrm{hyperHU}}_{\text{min}}=130$, respectively. (b) Corresponding manual gold standard. The whole tumour and the segmented hyper-dense region contours are displayed as dashed yellow and solid magenta lines, respectively.

Since hyper-dense regions are particularly heterogeneous due to interspersed foci of dense calcifications and non-calcified or less densely calcified tissue (see, for example, Fig. 5), only the sub-regions with the highest HU values would be detected in a single execution of the clustering algorithm. We overcame this problem by performing several executions of the fuzzy clustering with a C=2 procedure using the same cluster selection value ${\mathrm{hyperHU}}_{\text{min}}$ for each iteration (Fig. 2). More precisely, the clustering algorithm analyses the pixels that were not assigned to the high-intensity cluster $Y_{1}$ during the previous iteration until the current $v_{1}$ is lower than ${\mathrm{hyperHU}}_{\text{min}}$. In this manner, the hyper-dense component is identified by incrementally adding the regions that satisfy the criteria based on the cluster selection value ${\mathrm{hyperHU}}_{\text{min}}$. Therefore, we can explicitly deal with the heterogeneity of the hyper-dense tissues (i.e., calcifications or vessels). Fig. 5 shows an example of the incremental results achieved by three executions of the clustering procedure. Last, a morphological closing operation (by using a circular structuring element with a two-pixel radius) was applied to make the sub-region boundaries smoother.

### 3.2 Hypo-dense tissue segmentation

As shown in Fig. 3, the hypo-dense component segmentation relied on the binary mask yielded by the first phase. Indeed, the clustering algorithm was applied on the pixels not assigned to the hyper-dense region (i.e., this binary mask could be also completely 0-valued when no hyper-dense region was previously detected). The hypo-dense and the intermediately dense regions were segmented by using the fuzzy clustering with C=2. After its execution, if the minimum intensity centroid $v_{2}$ was lower than the maximum hypo-dense cluster selection value ${\mathrm{hypoHU}}_{\text{max}}$, the low-intensity cluster $Y_{2}$ was assigned as a hypo-dense region. To achieve a higher sensitivity in the identification of small hypo-dense regions, a fixed thresholding – using the well-established value of 20 HU for cystic/necrotic regions – was employed in the case of no detection *via* the proposed clustering-based pipeline.

Finally, the following morphological operations were performed to refine the sub-segmentation results [51]:

• a small-area removal operation, dealing with connected-components smaller than 0.08 cm${}^{2}$, to remove small regions not relevant for clinical purposes or radiomic applications;
• morphological closing (circular structuring element with two-pixel radius) to smooth the hypo-dense region boundaries;
• a hole-filling algorithm on the segmented hypo-dense region to remove possible holes due to local inhomogeneities.

## 4 Materials and evaluation methods

### 4.1 Patient dataset composition

The proposed framework segments the clinical CT scans of patients affected by (*i*) HGSOC and (*ii*) RCC. All the patients had been referred for clinically indicated CT scans by their clinical team. Both studies were approved by the local ethical review board. Written, informed consent to participate in this research was obtained from patients with ovarian cancer. For patients with RCC, the need for informed consent was waived.

All the analysed CT data are encoded in the 16-bit Digital Imaging and Communications in Medicine (DICOM) format. The dataset comprised axial CT scans acquired at multiple institutions by using scanners from three different vendors: General Electric Healthcare (Waukesha, WI, USA); Siemens Healthineers (Erlangen, Germany); and Toshiba Medical Systems (Tokyo, Japan). The main CT acquisition characteristics for the two datasets are reported in Table 1. Fig. S3 (in Supplementary Material) shows the volume distribution for the whole tumour, hyper-dense and hypo-dense components for the three considered tumour lesion locations. In all the cases, the volume distributions are right-skewed and present outliers, thus showing the intrinsic variability across the samples. Fig. S4 (in Supplementary Material) shows the variability of the Signal-to-Noise-Ratio (SNR), computed as ${\text{SNR}}_{\text{ROI(WholeTumour)}}=\frac{\mu_{\text{ROI(WholeTumour)}}}{\sigma_{\text{ROI(WholeTumour)}}}$, across the three tumour lesion locations analysed in this study.

**Table 1:** CT acquisition parameters of the HGSOC and RCC datasets.

Dataset	Peak voltage [kV]	Matrix size [pixels]	Slice thickness [mm]	Pixel spacing [mm]	
HGSOC	{100,120,130}	512 × 512	2.0–5.0	0.627–0.977	
RCC	{100,120,140}	512 × 512	{3.75,5.0}	0.586–0.965	

#### 4.1.1 High-grade serous ovarian carcinoma

CT scans of the abdomen and pelvis of 29 patients with HGSOC were included in this study. All ovarian cancers contained tumour of intermediate density together with either hypo-dense or hyper-dense portions, or both. We selected the most frequent and clinically relevant anatomic locations of HGSOC metastases, which are the pelvis and ovaries (Pelvic and Ovarian Disease, POD) and in the omentum. Overall, 26 and 10 POD and omental lesions, respectively, were considered. The total number of analysed CT slices was 965, where the average number of slices per lesion was 26.8±19.5 and 25.7±19.4 for POD and omental lesions, respectively. The average lesion volume was highly variable: 769.8±1068.7 cm${}^{3}$ and 290.1±435.4 cm${}^{3}$ for POD and omental lesions, respectively. More specifically, considering the tissue-specific Regions of Interest (ROIs), the number of hyper-dense (hypo-dense) regions was 15 (24) and 9 (7) for the POD and omentum, respectively.

#### 4.1.2 Renal cell carcinoma

The RCC dataset was composed of 10 patients with a total number of 152 analysed CT slices (average number of slices per lesion: 15.2±6.2). All the renal lesions considered in this study contained hypo-dense tissue components and only two revealed small calcifications (volume lower than 0.2 cm${}^{3}$).

The average volume of the lesions was 215.1±182.1 cm${}^{3}$. Whilst CT scans in patients with HGSOC were acquired during the portal venous phase, renal CT scans were acquired during the nephrographic phase, which involves a longer delay after the injection of intra-venous contrast agent.

### 4.2 Evaluation methodology

In this section, we describe the gold standard delineation strategy and the used region detection evaluation metrics.

#### 4.2.1 Gold standard delineation procedure

CT images were loaded into Microsoft Radiomics (project InnerEye,^1^ Microsoft, Redmond, WA, USA) and the entire POD, as well as any metastases in the omentum, were semi-automatically outlined in consensus by three readers: a medical doctor and Ph.D. student with 1.5 years of training and experience in cancer imaging (S.U.), a radiology registrar with five years of experience (L.B.), and a consultant radiologist with ten years of experience (R.W.) in general radiology and oncological imaging.

Hypo-dense areas that represented cystic or necrotic parts were identified visually and outlined separately. The same was done with the hyper-dense tumour portions that represented calcifications. We optimised window settings for the identification and semi-automatic segmentation of calcified tumour portions similar to the approach proposed in [52]. We measured the attenuation in the solid tumour part by manually placing an ROI${}_{\text{solid}}$ there. The mean of the HU in the ROI${}_{\text{solid}}$ was then used to estimate the optimal window level and width, respectively: ${\text{Window}}_{\text{level}}=\text{HU}\left({\text{ROI}}_{\text{solid}}\right)\cdot 2.68$ and ${\text{Window}}_{\text{width}}=\text{HU}\left({\text{ROI}}_{\text{solid}}\right)\cdot 3.1$.

**Fig. 6:**
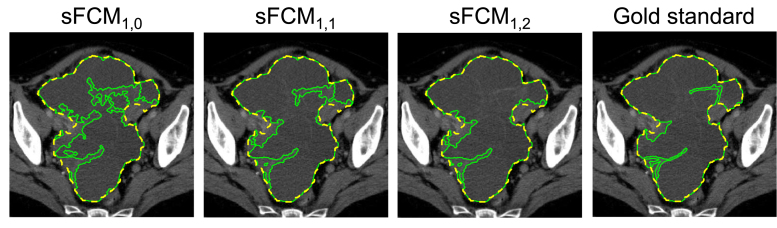
Influence of the weighting parameters p and q in the sFCM${}_{p,q}$ algorithm compared against the gold standard delineation. The whole tumour, hyper-dense, and hypo-dense region contours are displayed as dashed yellow, solid magenta and solid green lines, respectively.

**Fig. 7:**
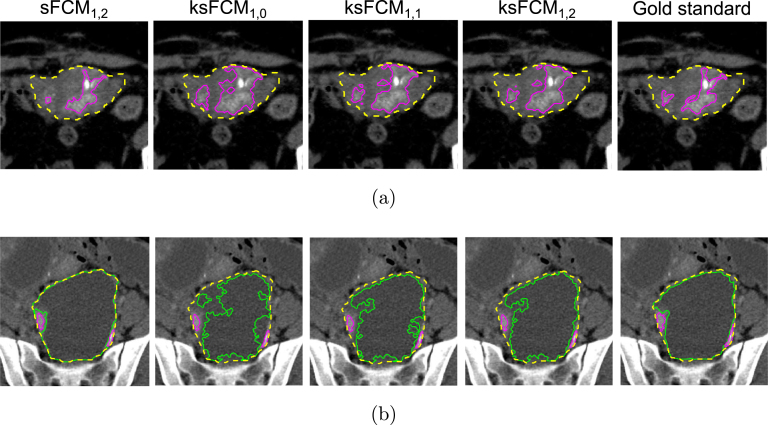
Example of results achieved by sFCM${}_{1,2}$ compared to ksFCM${}_{1,q}$ by varying the spatial component weight q∈{0,1,2}: (a) hyper-dense tissue segmentation; (b) hypo-dense tissue segmentation. In both cases, the gold standard delineation is shown at the right-most panel. The whole tumour, hyper-dense, and hypo-dense region contours are displayed as dashed yellow, solid magenta and solid green lines, respectively.

#### 4.2.2 Region detection and segmentation evaluation metrics

In order to assess the ability of the proposed method to correctly detect the slices with hypo-dense and hyper-dense components, we calculated the Area Under the Receiver Operating Characteristic Curve (AUROC).

For the quantitative evaluation of the image segmentation results achieved by the investigated computational methods, the automatically segmented CT images (S) were compared against the corresponding gold standard manual segmentation (G) using spatial overlap- and distance-based metrics [53], [54], [55]. Since our method analyses 2D CT images (mainly due to the slice thickness that may give rise to disconnected ROIs between adjacent slices), we calculated slice-wise metrics that were then averaged per patient. The segmentation evaluation metrics were computed separately for the hyper-dense and hypo-dense components. To achieve the goal of clinical and radiomic applications, a minimum area of 0.15 cm${}^{2}$ was set for the sub-region connected-components considered in the segmentation evaluation metrics calculation. In this manner, we decrease the effect on our assessment values caused by potentially created ROIs that consist of too-few pixels to be relevant for clinical or radiomic approaches. The used medical image segmentation evaluation metrics are described in Section S2 of the Supplementary Material.

The two-sided Wilcoxon signed rank test on paired DSC results [56] was performed (for each type of the segmented regions in a slice-wise fashion) with the null hypothesis that the samples come from continuous distributions with equal medians. In all the tests, a significance level of 0.05 was considered.

**Fig. 8:**
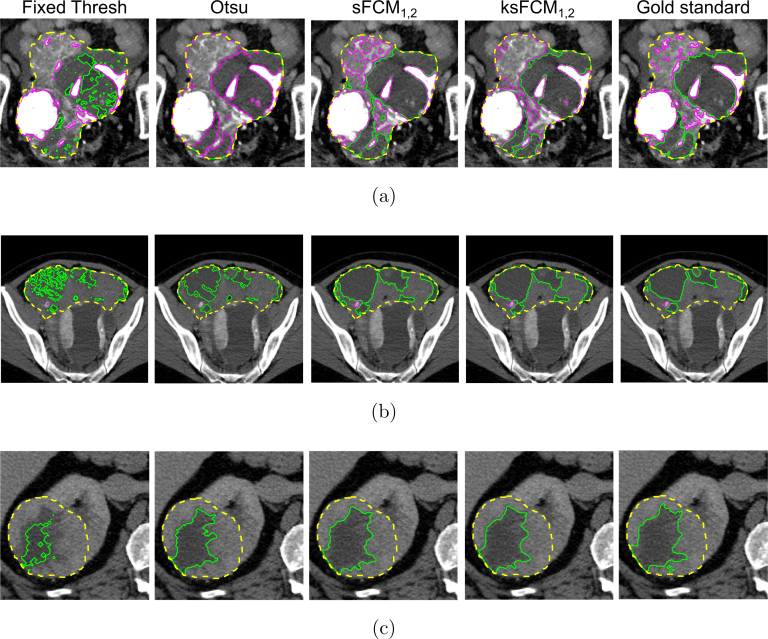
Segmentation results computed on the input CT images in Fig. 1. The whole tumour, hyper-dense, and hypo-dense region contours are displayed as dashed yellow, solid magenta and solid green lines, respectively.

#### 4.2.3 Competing methods

Since no existing literature work has addressed the tissue-specific sub-segmentation of the whole tumour burden on CT images so far, an experimental comparison of the proposed unsupervised FCM-based techniques was performed against the following segmentation approaches:

• fixed multi-threshold approach, which relies on clinically- established thresholds: pixels with values higher than 220 HU or lower than 20 HU are assigned to the hyper-dense and hypo-dense clusters, respectively. Relying on [57], [58], a threshold of 220 HU is generally used for aortic calcifications;
• two-stage Otsu method [59], which executes the same controls, based on the HU values for the inclusion in the hyper-dense and hypo-dense clusters, and post-processing steps;
• automatic selection of the number of regions based on cluster evaluation methods. Considering the crisp K-means clustering algorithm [60], K was estimated for each slice (aiming at a fine-grained control for finding the underlying tissue distribution). The tested heuristics were: the Caliński–Harabasz (CH) criterion [61]; the Davies–Bouldin (DB) criterion [62]; the silhouette criterion [63]; the gap statistics [64]. For all the techniques, the range of values used was K∈{1,2,3}. The automatic modified FCM cluster segmentation algorithm, proposed by Li and Shen [28], is unsuitable since the used cluster validity function, based on the fuzzy partitions (explicitly considering the cardinality of each cluster), might be highly affected by the ROI sizes and class imbalance.

## 5 Experimental results

This section presents the experimental results achieved by the proposed computational framework, by showing both graphical examples and quantitative evaluation metrics.

Fig. 6 shows an example of hypo-dense tissue segmentation results by varying the weighting parameters, p and q, in the case of the sFCM algorithm. It is worth noting that the higher the spatial weighting q, the more connected the segmented areas; this applies especially in the case of highly heterogeneous hypo-dense tissue components.

Furthermore, two examples of the implemented ksFCM versions (with various values of the q parameter) compared against the sFCM${}_{1,2}$ for hyper-dense and hypo-dense tissue segmentation are depicted in Fig. 7, Fig. 7, respectively. In both cases, the introduction of the spatial context also incrementally improves the segmentation results also for the kernelised version. However, the delineations of all the ksFCM are less accurate than those achieved by sFCM${}_{1,2}$.

Fig. 8 shows the results achieved by the implemented methods on the CT images in Fig. 1. For higher visibility, we display only sFCM${}_{1,2}$ and ksFCM${}_{1,2}$ results (achieving the best overall qualitative and quantitative performance among the tested p and q values), along with the fixed thresholding and two-stage Otsu methods. The fixed thresholding, as well as the Otsu method, tends to under-estimate the segmented regions. In particular, in the case of large inhomogeneous hypo-dense components, the segmentation might present many disconnected and spurious areas. In addition, some small calcifications could be missed. Furthermore, the tested two-stage Otsu approach could fail in the case of lesions with highly mixed tissue components (Fig. 8, Fig. 8). With regard to unsupervised fuzzy clustering methods, sFCM${}_{1,2}$ generally yields more accurate segmentation results than ksFCM${}_{1,2}$; Fig. 8(a), in particular, shows the high ability to detect diffuse calcified tissue, as well as small details in the hypo-dense component.

**Fig. 9:**
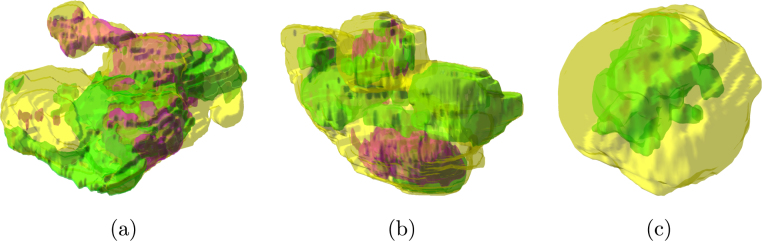
Three-dimensional reconstruction of the segmented ROIs (green and magenta volumetric models for the hyper- and hypo-dense components, respectively) in their actual location with respect to the enclosing whole tumour (transparent yellow surface): (a) POD, (b) omental lesion, (c) RCC. The transparent surfaces are rendered with alpha blending (α=0.40).

To better demonstrate how the tumoural tissue components appear intertwined, Fig. 9 shows three examples of three-dimensional rendering of the ROIs, allowing us to display their actual locations in the whole tumour (represented by means of the enclosing transparent yellow surface).

**Table 2:** AUROC achieved by the compared tissue-specific CT image sub-segmentation methods on the HGSOC (POD and omental lesions) and RCC datasets.

Method	HGSOC (POD)		HGSOC (omentum)		RCC	
	Hyper-dense	Hypo-dense	Hyper-dense	Hypo-dense	Hyper-dense	Hypo-dense
Fixed thresholding	0.781	0.534	0.674	0.573	0.667	0.771
Two-stage Otsu	0.682	0.689	0.623	0.716	0.500	0.802
Silhouette ＋ K-means	0.694	0.578	0.572	0.589	0.466	0.739
CH ＋ K-means	0.471	0.501	0.499	0.492	0.333	0.500
DB ＋ K-means	0.481	0.507	0.528	0.535	0.282	0.553
Gap ＋ K-means	0.628	0.754	0.519	0.599	0.490	0.719
sFCM${}_{1,0}$	0.901	0.987	0.937	0.987	0.943	0.981
sFCM${}_{1,1}$	0.901	0.987	0.937	0.987	0.943	0.981
sFCM${}_{1,2}$	0.901	0.987	0.937	0.987	0.943	0.981
ksFCM${}_{1,0}$	0.917	0.982	0.937	0.981	0.943	0.981
ksFCM${}_{1,1}$	0.901	0.982	0.937	0.981	0.943	0.981
ksFCM${}_{1,2}$	0.901	0.987	0.937	0.987	0.943	0.981

### 5.1 Region detection and segmentation results

Table 2 shows the AUROC for evaluating the specificity and sensitivity of the performance of hyper-dense and hypo-dense region detection. The first experimental finding was that the fixed thresholding and the two-stage Otsu method do not perform adequately. Similarly, the automatic strategies for the selection of the number of clusters for the K-means algorithm showed a poor performance. This could be observed particularly in HGSOC due to the higher prevalence of hyper-dense and hypo-dense components, compared to RCC. The proposed two-stage approach based on unsupervised fuzzy clustering achieves excellent detection performance by overcoming the need for the *a priori* number of clusters. On the contrary, the same two-stage approach employing the Otsu method in place of the fuzzy clustering algorithms did not achieve a comparable performance.

**Fig. 10:**
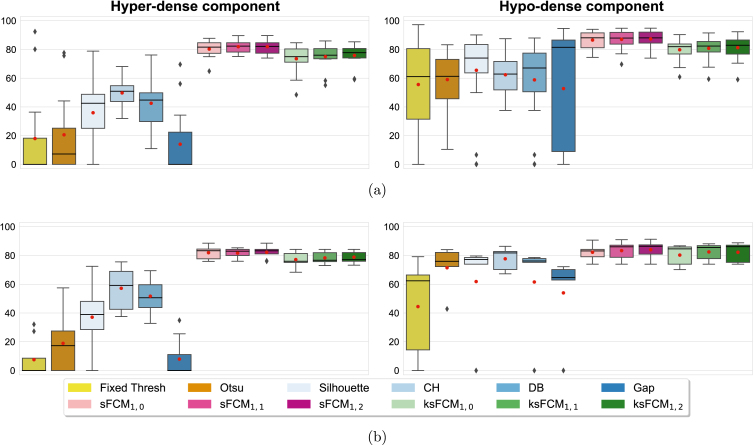
DSC values of the tissue-specific sub-segmentation results for the (a) POD and (b) omental lesions on the HGSOC CT datasets.

**Fig. 11:**
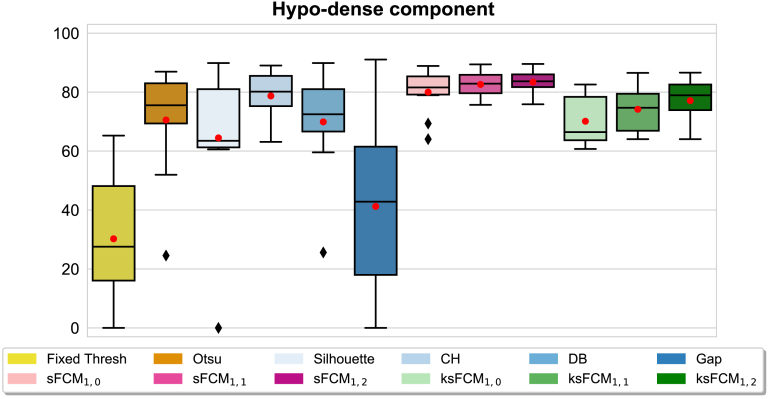
DSC values of the tissue-specific sub-segmentation results for the kidney lesions on the RCC CT datasets.

Regarding the segmentation evaluation metrics described in Section 4.2.2, for conciseness and clarity, we report only the DSC values in what follows. Figs. 10(a), 10(b), and 11 plot the distribution of the DSC values achieved on the POD, omental, and RCC lesions, respectively. All the boxplots display a black solid line and a red circular marker that denote the median and mean values, respectively. The whisker value is set to 1.5 in all cases and the outliers are displayed as black diamonds. The legend box at the bottom denotes the investigated classes of methods with different colour palettes. For completeness, the results of the other metrics are provided in the Supplementary Material (Figs. S5–S14) and are used to support the result analysis.

 The fixed thresholding and two-stage Otsu methods obtain low DSC values, because they typically under-estimate the segmented regions (low sensitivity and high specificity values). In accordance with the AUROC values in Table 2, the highly variable DSC results, obtained by the four tested heuristics for the K-means algorithm [60], point out the difficulty of selecting the correct number of clusters; among these strategies, the CH criterion [61] achieved the overall best performance whilst the gap statistics [64] showed highly unreliable results.

In general, the unsupervised fuzzy clustering configuration with p=1 and q=2 outperformed the other configurations for both sFCM and ksFCM. The introduction of the spatial information provided significant benefits over the classic FCM algorithm. However, sFCM${}_{1,2}$ overall achieved higher performance than ksFCM${}_{1,2}$. In particular, sFCM${}_{1,2}$ significantly outperformed ksFCM${}_{1,2}$ in the case of POD lesions ($p=2.282\times 10^{-4}$ and $p=1.483\times 10^{-49}$ for hyper-dense and hypo-dense DSC values, respectively), as well as RCC lesions (p=0.0011) for hypo-dense DSC values, respectively). In more detail, all the ksFCM configurations often fail on noisy images by disconnecting areas with local inhomogeneities, such as large heterogeneous hypo-dense regions. The low presence of hyper-dense components and the large hypo-dense areas in the case of the RCC dataset (Fig. 11), compared to the HGSOC lesions (Fig. 10, Fig. 10), can explain the typically better performance of the K-means clustering using the heuristics for the number of cluster selection.

### 5.2 Computational performance

The computational performance, in terms of processing time and memory consumption, was measured. The execution times were computed by means of the tic and toc stopwatch timer functions. Moreover, by relying on the size of the variables allocated in the MatLab workspace, we estimated the amount of memory required by the investigated methods. Aiming at a practical use case, we selected a patient with HGSOC in which there was a large pelvic lesion (4690.6 cm${}^{3}$), with large cystic components, extended across 51 CT slices. (See Table 3.)

**Table 3:** Computational performance achieved by the implemented methods in terms of processing times and memory consumption for the HGSOC CT scan (considering a large POD lesion) selected as a practical use case.

Method	Processing time [s]	Memory [GB]
Fixed thresholding	0.1345	1.0185
Two-stage Otsu	1.8046	1.0170
Silhouette ＋ K-means	$1.4200\times 10^{3}$ (=23.667 mins)	1.0498
CH ＋ K-means	17.9152	1.0498
DB ＋ K-means	18.1542	1.5718
Gap ＋ K-means	$2.0796\times 10^{3}$ (=34.66 mins)	1.0478
sFCM${}_{1,0}$	18.4728	1.0246
sFCM${}_{1,1}$	25.3067	1.0246
sFCM${}_{1,2}$	24.7146	1.0246
ksFCM${}_{1,0}$	31.4829	1.0246
ksFCM${}_{1,1}$	52.5809	1.0246
ksFCM${}_{1,2}$	51.1077	1.0246

The fixed thresholding was the most time-efficient approach, along with the two-stage Otsu method. Among the heuristics for the selection of the number of clusters for the K-means clustering, the silhouette and the gap statistics are particularly demanding with respect to the CH and DB criteria (whose processing times are in line with sFCM${}_{1,0}$). Considering the unsupervised fuzzy clustering implementations, the processing time of sFCM increases with the introduction of the spatial function (see Supplementary Material), even though the computational overhead is mitigated for q=1 and q=2. Comparing ksFCM${}_{1,0}$ and sFCM${}_{1,0}$, an increase in processing times is appreciable due to the transformation of all the input pixel values into the feature space by means of the GRBF kernel). Interestingly, the trend regarding the spatial version, by varying q in {1,2,3}, is valid also for the kernelised implementations.

With reference to the computational complexity, since our framework uses only the HU values (i.e., D=1) and two clusters (i.e., C=2), the overall time and memory requirements are suitable for nearly real-time performance. Indeed, all the sFCM and ksFCM versions have a linear O(N) and quadratic $O\left(N^{2}\right)$ time complexity, respectively. Interestingly, C=2 implies that the membership matrix U can be stored using 2×N double-precision floating-point numbers (i.e., 8 bytes in MatLab). Regarding the scalability, since the clustering algorithm is applied for each 2D slice, the implementation scales with the number of slices composing the whole tumour burden. Therefore, a distributed computing paradigm can be leveraged to offload onto multiple CPU cores the independent computations concerning the different slices to segment [65].

## 6 Discussion and conclusion

In this work, we proposed an intelligent tissue-specific sub- segmentation framework based on unsupervised fuzzy clustering techniques, which allows for clinically interpretable and radiomics-oriented results. Our novel approach, leveraging a two-stage *divide-et-impera* strategy, accurately and efficiently detects and delineates the hyper-dense and hypo-dense components in heterogeneous tumours, thus overcoming the limitations imposed by the automatic selection of the number of clusters required by partitional clustering techniques. We tested our approach on two datasets comprising highly heterogeneous tumours, namely, HGSOC and RCC. Both detection and segmentation performance with regard to tissue components – in terms of AUROC and overlap-/distance-based evaluation metrics, respectively – showed superiority over the existing methods (namely, fixed thresholding, two-stage Otsu method, automatic clusters number selection heuristics for the K-means clustering algorithm). More specifically, sFCM${}_{1,2}$ generally outperformed the other clustering configurations, even when compared to the kernelised versions, in particular. Therefore, the proposed framework could be suitably transferred into biomedical research environments (without requiring any training/set-up phases) for robust radiomic biomarker development [23], [66].

From a clinical perspective, the proposed computational framework, yielding interpretable results, might represent a reliable and feasible solution, since it obtains a DSC higher than 70% overall, which is generally regarded as a satisfactory level of agreement between two segmentations (i.e., manual and automated delineations) in clinical applications [67], [68]. The accurate segmentation performance achieved by our two-stage framework, in terms of the DSC metric, was confirmed by a good balance of the sensitivity and specificity values. The experimental findings provided by the overlap-based metrics are endorsed by the distance-based metrics that consider the delineated region boundaries. Generally, sFCM achieved more accurate results than ksFCM, consistent with the results presented in [31], where sFCM (with p=1 and q=2) significantly outperformed the K-means, classic FCM, and the kernelised version in brain MRI tissue segmentation.

This single-lesion-focused study on intra-tumoural heterogeneity could be extended to multiple sites to evaluate intra-/inter-tumoural heterogeneity, especially in the case of HGSOC, which typically comprises a heterogeneous mixture of solid and cystic tissue and has frequently metastasised to multiple anatomic locations when diagnosed [69], [70]. With regard to RCC, the macroscopic heterogeneity visible on CT is typically caused by necrosis, haemorrhage, and cystic parts [71]. These typical morphological characteristics even allow for a cancer classification based on the appearance of a tumour on CT [72]. CT-based texture feature computation on intermediately dense tumour tissue alone was shown to be effective in the literature: Takahashi et al. [73] drew the largest possible circular ROI avoiding calcifications, whilst Lend et al. [74] manually excluded calcifications and cystic/necrotic parts from the whole tumour. The implementation of our approach for tissue sub-segmentation into a clinical research workflow, which aims at establishing radiomic biomarkers, might allow us to evaluate tissue-type-specific radiomics more extensively against whole-tumour radiomics. Since highly proliferative and aggressive tumour portions are frequently found in solid, non-calcified areas of ovarian cancer [10], radiomics specifically computed for these areas might convey more relevant predictive and prognostic information than global tumour radiomics. Another potential field of application is the clinical radiological setting, where treatment response is commonly assessed based on changes in the overall diameters of tumours [11] whilst this simplification disregards differential changes in solid versus cystic tumour components [75], [76]. An automated and reliable approach for the sub-segmentation of tumour sub-regions, as demonstrated here, might allow for more specific response assessment to be first evaluated and subsequently integrated into clinical research environments. Due to the interpretability of the results obtained using our proposed method, clinicians might be more amenable to the implementation of such a tool for clinical purposes compared to less interpretable “black box” approaches [77]. Potential areas of further investigation might regard the integration with circulating biomarkers, where CA125, which is an established clinical biomarker used for disease detection and monitoring in HGSOC [78], as well as circulating tumour cells and cell free tumour DNA in plasma, which are currently evaluated in translational oncological studies [79].

One of the limitations of this study is the continued requirement for relatively labour-intensive and time-consuming manual delineation of tumours and the inherent user-dependence [80]. Convolutional Neural Networks hold the potential to overcome this necessity when exploited to develop a fully automated segmentation approach for combined whole tumour detection and segmentation [81], which could be integrated with our unsupervised tissue-specific sub-segmentation pipeline. However, developing such a comprehensive framework requires large-scale annotated datasets for training/testing and was beyond the scope of this study, but might be a goal for developing the proposed method further. Another limitation is the relatively small number of patients included in this study. However, the large size of some of the selected lesions, which extended over 80 CT slices, also allowed the 2D clustering approach to be validated on a remarkably higher number of images (1117 in total) than the number of patients might suggest. In conclusion, we were able to show the effectiveness of the proposed approach and its advantages compared to the investigated competing methods on both HGSOC and RCC datasets.

## Declaration of Competing Interest

No author associated with this paper has disclosed any potential or pertinent conflicts which may be perceived to have impending conflict with this work. For full disclosure statements refer to https://doi.org/10.1016/j.compbiomed.2020.103751.

## Appendix A Supplementary data

MMC S1 .

## Footnotes

1 https://www.microsoft.com/en-us/research/project/medical-image-analysis/.
