# Supplementary material for: Tissue-specific and interpretable sub-segmentation of whole tumour burden on CT images by unsupervised fuzzy clustering
Source: Comput Biol Med. 2020 May;120:103751. doi: 10.1016/j.compbiomed.2020.103751 (PMC7248575; doi:10.1016/j.compbiomed.2020.103751)
Supplement: MMC S1 — . [file mmc1.zip › SupplementaryMaterial.pdf]

Supplementary Material

Tissue-specific and Interpretable Sub-segmentation of Whole Tumour Burden on CT Images by Unsupervised Fuzzy Clustering

Leonardo Rundo, Lucian Beer, Stephan Ursprung,

Paula Martin-Gonzalez, Florian Markowetz, James Brenton,

Mireia Crispin-Ortuzar, Evis Sala, Ramona Woitek

S1. Unsupervised fuzzy clustering techniques: mathematical formulation

This section explains the devised unsupervised fuzzy clustering framework designed to unify the classic, spatial, and kernelised versions of the Fuzzy C-Means (FCM) method [1, 2].

S1.1. The Fuzzy C-Means clustering algorithm

The FCM algorithm [1, 2] is a partitional clustering technique that minimises the intra-cluster variance, as well as maximises the inter-cluster variance, in terms of a distance metric between the feature vectors [3]. Given an input data space $\Omega = \mathbb{R}^D$, this unsupervised technique optimises the intrinsic partitioning of an unlabelled dataset $\mathcal{X} = \{\mathbf{x}_1, \mathbf{x}_2, \ldots, \mathbf{x}_N\}$ composed of N feature vectors, which denote data samples $\mathbf{x}_k \in \mathbb{R}^D$ $(k = 1, 2, \ldots, N)$ belonging to a D-dimensional Euclidean space, into exactly C clusters (i.e., non-empty partitions of the input dataset), as introduced in [1, 2]. An input dataset is partitioned into groups (i.e., regions in image segmentation tasks), and each of them is identified by a centroid. Distance metrics are used to group data into clusters of similar types and the number of clusters is assumed to be known *a priori* [4]. These feature vectors can be conveniently represented by a data matrix, $\mathbf{X} \in \mathbb{R}^{N \times D}$, with N rows containing the input D-dimensional feature vectors. Formally, a partition $\mathcal{P}$ is defined as a set family $\mathcal{P} = \{\mathcal{Y}_1, \mathcal{Y}_2, \ldots, \mathcal{Y}_C\}$.

In the case of the crisp K-means algorithm [5], the clusters must be a proper crisp subset of $\mathcal{X}$ $(\varnothing \subset \mathcal{Y}_i \subseteq \mathcal{X}, \forall i)$ and their set union must reconstruct the whole dataset $(\bigcup_{i=1}^{C} \mathcal{Y}_i = \mathcal{X})$. Moreover, the clusters are mutually

exclusive ($\mathcal{Y}_i \cap \mathcal{Y}_j = \varnothing, \forall i \neq j$), i.e., each feature vector can belong to only one group. In contrast to the K-means algorithm [6], where each feature vector can be assigned to only one group, the FCM algorithm defines a fuzzy partition, $\mathcal{P} = \{\mathcal{Y}_1, Y_2, \ldots, \mathcal{Y}_C\}$, using a fuzzy set family [7].

Formally, the FCM scheme allows for partial membership to multiple classes [8] with varying degrees [9]. Accordingly, the membership matrix $\mathbf{U} \in \mathbb{R}^{C \times N}$ denotes a fuzzy C-partition of the set $\mathcal{X}$ using the C membership functions $\mu_i : \mathcal{X} \to [0,1]$, with values $u_{ik} \triangleq \mu_i(\mathbf{x}_k) \in [0,1]$ representing the similarity membership of each element $\mathbf{x}_k$ to the i-th fuzzy set (i.e., the cluster $\mathcal{Y}_i$) and must meet the following conditions:

$$\begin{cases} 0 \leq u_{ik} \leq 1 & \\ \sum\limits_{i=1}^{C} u_{ik} = 1, & \forall k \in \{1, \ldots, N\} \\ 0 < \sum\limits_{k=1}^{N} u_{ik} < N, & \forall i \in \{1, \ldots, C\} \end{cases} . \tag{1}$$

With this notation, the sets of all hard and fuzzy C-partitions of the input dataset $\mathcal{X}$ can simply be defined by:

$$\mathcal{M}_{\text{hard}} = \left\{ \mathbf{U} \in \mathbb{R}^{C \times N} : u_{ik} \in \{0,1\} \right\}, \tag{2}$$

$$\mathcal{M}_{\text{fuzzy}} = \left\{ \mathbf{U} \in \mathbb{R}^{C \times N} : u_{ik} \in [0,1] \right\}, \tag{3}$$

where $\mathcal{M}_{\text{fuzzy}}$ enhances the information conveyed by $\mathcal{M}_{\text{hard}}$, because it allows for more flexibility [10], as well as more information is kept from the original data compared to the crisp K-Means approach [11].

Let $\mathcal{V} = \{\mathbf{v}_1, \mathbf{v}_2, \ldots, \mathbf{v}_C\}$ be a set of D-dimensional prototype vectors,

called centroids that are associated with the C clusters. The membership function values are assigned to each feature vector in the data matrix $\mathbf{X}$ according to the relative distance of each feature vector $\mathbf{x}_k$ from the centroids in $\mathcal{V}$. Thus, this clustering algorithm aims to optimise the objective function in Eq. (4), representing a generalised least-squares error problem:

$$\mathcal{J}_{\mathrm{FCM}}(\mathbf{U}, \mathcal{V}; \mathbf{X}) = \sum_{i=1}^{C} \sum_{k=1}^{N} (u_{ik})^m \cdot d_{ik}, \tag{4}$$

where:

- $m \in [1, \infty)$ is the fuzzification constant that weighs the fuzziness of the classification process. If $m = 1$, the FCM algorithm approximates the crisp K-means version. The role of the weighting exponent m in the FCM model was systematically analysed in [12], where the authors suggested that the best choice for m is in the interval $[1.5, 2.5]$, and its mean value $m = 2$ is typically used;

- $d_{ik} = \|\mathbf{x}_k - \mathbf{v}_i\|^2$ is the squared distance between the elements $\mathbf{x}_k$ and $\mathbf{v}_i$, computed by means of an induced norm, $\|\cdot\|$ on $\mathbb{R}^D$ (usually the Euclidean ℓ_2 norm).

Therefore, the data matrix $\mathbf{X}$ is partitioned by iteratively searching for the optimal fuzzy partition $\mathcal{P}$, denoted by the pair $(\hat{\mathbf{U}}, \hat{\mathcal{V}})$, which minimises the objective function $\mathcal{J}_m$ by means of a local optimisation technique. During the t-th iteration, each centroid $\hat{\mathbf{v}}_i \in \hat{\mathcal{V}}$ is updated by computing the mean

of all points, weighted by their degree of belonging to the i-th cluster:

$$\hat{\mathbf{v}}_i = \frac{\sum_{j=1}^{N} (\hat{u}_{ij})^m \mathbf{x}_j}{\sum_{j=1}^{N} (\hat{u}_{ij})^m}. \tag{5}$$

Similarly, Eq. (6) updates all the elements of the membership matrix $\hat{\mathbf{U}}$ considering the centroids $\hat{\mathbf{v}}_i \in \hat{\mathcal{V}}$ estimated, at the t-th iteration, *via* Eq. (5):

$$\hat{u}_{ik} = \left[\sum_{j=1}^{C} \left(\frac{\|\mathbf{x}_k - \hat{\mathbf{v}}_i\|}{\|\mathbf{x}_k - \hat{\mathbf{v}}_j\|} \right)^{\frac{2}{m-1}} \right]^{-1}, \text{with } m > 1 \text{ and } \mathbf{x}_k \neq \hat{\mathbf{v}}_j \ \forall j, k. \tag{6}$$

Afterwards, each object $\mathbf{x}_k$ is compared with the elements of the centroid vector and is then mapped onto the nearest cluster. Convergence conditions can be defined by considering either the minimum improvement in objective function $\mathcal{J}_{\text{FCM}}$ between two consecutive iterations or the Frobenius norm of the difference of the matrices $\hat{\mathbf{U}}^{(t+1)}$ and $\hat{\mathbf{U}}^{(t)}$. The iterative procedure ends when the convergence condition is less than a fixed tolerance value ε_{tol} or the maximum number of allowed iterations $T_{\max}$ is achieved. The initial partitions in $\mathbf{U}$ are randomly generated by imposing that the sum of the elements of each column is equal to one. For all the implemented fuzzy clustering methods, we used $\varepsilon_{\text{tol}} = 10^{-5}$ and $T_{\max} = 100$ in the criterion that compares the value of the objective function between two consecutive iterations.

S1.2. Spatial FCM clustering

The classic FCM clustering algorithm does not take into account any spatial relationship among pixels since all the samples are used as disperse and

independent points, making it sensitive to noise and other imaging artefacts [13]. Accordingly, the integration of spatial information might be beneficial.

The earliest version of the spatial FCM (sFCM) algorithm was formulated as a regularisation term to penalise the FCM objective function in Eq. (4)—which relies on pixel values alone regardless of their location—by conveying spatial information and constraining the behaviour of the membership functions [9], similarly to methods used in the regularisation and Markov Random Field (MRF) theory [14, 15]. Thus, a fuzzy local similarity measure is integrated into its objective function, aimed at decreasing the sensitivity to noise, as well as preserving image details [16]. Accordingly, the objective function of the robust Fuzzy Local Information C-Means (FLICM) algorithm estimating spatially smooth membership functions [17] aims at:

$$\mathcal{J}_{\text{FLICM}}(\mathbf{U}, \mathcal{V}; \mathbf{X}) = \sum_{i=1}^{C} \sum_{k=1}^{N} (u_{ik})^m \cdot d_{ik} + \mathcal{H}_{ik}, \tag{7}$$

where the fuzzy factor $\mathcal{H}_{ik}$ acts as a regulariser:

$$\mathcal{H}_{ik} = \sum_{\substack{j \in \mathcal{N}(\mathbf{x}_k) \\ k \neq j}} \frac{1}{1 + d_{jk}} (1 - u_{ij})^m \cdot \|\mathbf{x}_j - \mathbf{v}_i\|^2, \tag{8}$$

considering the k-th pixel as the centre of the local window defined by the neighbourhood $\mathcal{N}(\mathbf{x}_k)$, i.e., a squared window of $\omega \times \omega$ pixels, whilst i denotes the currently analysed cluster.

The sFCM, alternatively introduced by Chuang *et al.* [18], enables the retention of the same formulation and objective function as the classic FCM algorithm described in Section S1.1, just by modifying the update rules with the local spatial content in the image. With the goal of exploiting the contextual information, a spatial function is defined as [4, 18]:

$$h_{ik} = \sum_{j \in \mathcal{N}(\mathbf{x}_k)} \hat{u}_{ij}. \tag{9}$$

Basically, just like the membership function, the spatial function h_{ik} represents the membership degree of pixel $\mathbf{x}_i$ belonging to the j-th cluster: the higher its values, the larger the number of neighbours that will belong to the same clusters. The spatial function modifies the membership function of a pixel according to the membership statistics of its neighbours as follows:

$$\hat{u}'_{ik} = \frac{\hat{u}^p_{ik} \cdot h^q_{ik}}{\sum_{j=1}^{C} \hat{u}^p_{jk} \cdot h^q_{jk}}, \tag{10}$$

where $\hat{u}_{ik}$ is computed according to Eq. (6). Finally, as in the case of the classic FCM algorithm, the centroid vector is updated according to Eq. (5) by just using $\hat{u}'_{ik}$ in place of $\hat{u}_{ik}$. Hereafter, in compliance with the notation introduced in [18], we denote the sFCM with the control parameters p and q as sFCM$_{p,q}$. According to the literature [18, 13], we tested $p = 1$ and $q \in \{0, 1, 2\}$.

The incorporation of the spatial component considerably improves the performance: (i) in a homogeneous region, the spatial functions emphasise the original membership, so the clustering results are not affected; (ii) in noisy regions, spurious blobs or misclassified pixels may be corrected. According to [18], in all the tests, a local window with $\omega = 5$ was used.

S1.3. Kernelised FCM clustering

The metric used in the objective function of the FCM versions described in Sections S1.1 and S1.2 is still the Euclidean distance, as in FCM. However,

the use of the ℓ_2 norm might lead to non-robust results on the segmentation of an image corrupted by noise, outliers, and other imaging artefacts. The kernelised methods let us generalise distance-based algorithms to operate in feature spaces, usually non-linearly related to the input space. This can be performed by identifying a class of kernels that can be represented as norm-based distances in Hilbert spaces [19]. The fundamental idea of the kernel method is to first transform the original low-dimensional inner-product input space into a higher (possibly infinite) dimensional feature space by using a non-linear mapping [20]. Quite interestingly, kernelised methods are suitable for clustering algorithms [16].

Mathematically, a kernel function ($\mathcal{K} : \Omega \times \Omega \to \mathbb{R}$) enables operations in a high-dimensional feature space Φ without the need to compute the coordinates of the input data Ω in that space, but rather, by simply computing the inner products between the images of all pairs of data in the feature space. This strategy is typically called the "kernel trick", performed by means of an implicit non-linear mapping ($\phi : \Omega \to \Phi$) that satisfies Eq. (11):

$$\mathcal{K}(\mathbf{x}, \mathbf{y}) = \langle \phi(\mathbf{x}), \phi(\mathbf{y}) \rangle_\Phi, \tag{11}$$

where $\langle \cdot, \cdot \rangle_\Phi$ denotes the inner product in the feature space Φ.

The objective function of the kernelised version of the FCM algorithm is defined in Eq. (12):

$$\mathcal{J}_{\text{kFCM}}(\mathbf{U}, \mathcal{V}; \mathbf{X}) = \sum_{i=1}^{C} \sum_{k=1}^{N} (u_{ik})^m \cdot \|\phi(\mathbf{x}_i) - \phi(\mathbf{v}_k)\|^2, \tag{12}$$

where, because of the kernel substitution trick, the distance term can be

rewritten as:

$$\|\phi(\mathbf{x}_i) - \phi(\mathbf{v}_k)\|^2 = (\phi(\mathbf{x}_i) - \phi(\mathbf{v}_k))^\top(\phi(\mathbf{x}_i) - \phi(\mathbf{v}_k)) = \\ = \mathcal{K}(\mathbf{x}_i, \mathbf{x}_i) + \mathcal{K}(\mathbf{v}_k, \mathbf{v}_k) - 2\mathcal{K}(\mathbf{x}_i, \mathbf{v}_k). \tag{13}$$

By using this mapping technique, the interpretability of the clustering results is maintained since the centroids of the clusters are still represented in the original input space Ω, whilst the clustering procedure is performed in the feature space Φ. More specifically, the update formulation in Eqs. (6) and (5) becomes, respectively [9]:

$$\hat{u}_{ik} = \frac{1 - \mathcal{K}(\mathbf{x}_i, \mathbf{v}_k)^{-1/(m-1)}}{\sum_{j=1}^{C} 1 - \mathcal{K}(\mathbf{x}_i, \mathbf{v}_j)^{-1/(m-1)}}, \tag{14}$$

$$\hat{\mathbf{v}}_i = \frac{\sum_{j=1}^{N} (\hat{u}_{ij})^m \mathcal{K}(\mathbf{x}_j, \mathbf{v}_i)\mathbf{x}_j}{\sum_{j=1}^{N} (\hat{u}_{ij})^m \mathcal{K}(\mathbf{x}_j, \mathbf{v}_i)}. \tag{15}$$

Generally, the Gaussian Radial Basis Function (GRBF) kernel is adopted:

$$\mathcal{K}(\mathbf{x}, \mathbf{y}) = e^{-\frac{\|\mathbf{x}-\mathbf{y}\|^2}{2\sigma^2}}, \tag{16}$$

where σ denotes the kernel width. Since σ is a particularly sensitive parameter we relied upon [16], where an adaptive strategy is used to determine the kernel parameters by using the fast bandwidth selection rule in Eq. (17), based on the distance variance of all data points in the collection:

$$\sigma = \sqrt{\frac{1}{N-1}\sum_{i=1}^{N}\left(d_i - \bar{d}\right)^2}, \tag{17}$$

where $d_i = \|\mathbf{x}_i - \bar{\mathbf{x}}\|$ is the distance from the grey-scale of the i-th pixel to the grey-scale average of all pixels and $\bar{d}$ is the average of all distances d_i.

The kernel-based spatial FCM model used does not adopt the dual representation for each centroid, i.e., a linear combination of all given dataset samples, but directly transforms all the centroids in the original space, together with given data samples, into high-dimensional feature space with an implicit mapping [20]. Such a direct transformation allows for inducing a class of robust non-Euclidean distance measures when robust kernels are exploited. At the same time, the computational simplicity and efficiency of the FCM are maintained.

To perform a fair comparison independent of centroid initialisation, our kernelised sFCM (ksFCM) version exploited the formulation adopted by the classic FCM algorithm explained in Eq. (5) in the original space, starting from the same random $\mathbf{U}$ matrix initialisation, for result repeatability. Indeed, the update formulation for the kernel-based method requires the transformed samples in the feature space, by involving the computation of the kernel function with respect to the current centroid set $\mathcal{V}$.

S2. Medical image segmentation evaluation metrics

For the quantitative evaluation of the image segmentation results achieved by the investigated computational methods, the automatically segmented CT images (S) were compared against the corresponding gold standard manual segmentation (G) using spatial overlap- and distance-based metrics [21, 22, 23].

S2.1. Overlap-based metrics

These metrics quantify the spatial overlap between the segmented ROIs. Let the true-positives be TP $= \mathsf{S} \cap \mathsf{G}$, false-negatives be FN $= \mathsf{G} - \mathsf{S}$, false-positives be FP $= \mathsf{S} - \mathsf{G}$, and true-negatives be TN $= \mathcal{I} - \mathsf{G} - \mathsf{S}$ (where $\mathcal{I}_{\mathrm{ROI}}$ denotes the input ROI image). In what follows, we denote the cardinality of the pixels that belong to a region A as $|\mathsf{A}|$.

- The Dice similarity coefficient [24] is the most used measure in medical image segmentation to compare the overlap of two regions:

$$\mathrm{DSC} = \frac{2 \cdot |\mathrm{TP}|}{|\mathsf{S}| + |\mathsf{G}|} \cdot 100. \tag{18}$$

- Sensitivity measures the correct detection ratio of true-positives:

$$\mathrm{SEN} = \frac{|\mathrm{TP}|}{|\mathrm{TP}| + |\mathrm{FN}|} \cdot 100. \tag{19}$$

- Specificity usually measures the true-negative detection ratio:

$$\mathrm{SPC}_{\mathrm{TNR}} = \frac{|\mathrm{TN}|}{|\mathrm{TN}| + |\mathrm{FP}|} \cdot 100; \tag{20}$$

  however, this formulation is ineffective when data are unbalanced (i.e., the ROI is much smaller than the whole image). Consequently, we use the following definition:

$$\mathrm{SPC} = \left(1 - \frac{|\mathrm{FP}|}{|\mathsf{S}|}\right) \cdot 100. \tag{21}$$

S2.2. Distance-based metrics

Since precise boundary tracing plays an important role in clinical practice, overlap-based metrics have limitations in evaluating segmented images. In

order to measure the distance between the two ROI boundaries, distance-based metrics can be considered. Let the manual contour $\mathcal{G}$ consist of a set of vertices $\{\mathbf{g}_a : a = 1, 2, \ldots, A\}$ and the automatically-generated contour $\mathcal{S}$ consist of a set of vertices $\{\mathbf{s}_b : b = 1, 2, \ldots, B\}$. We calculate the absolute distance between an arbitrary element, $\mathbf{s}_b \in \mathcal{S}$, and all the vertices in $\mathcal{G}$ as follows:

$$d(\mathbf{s}_b, \mathcal{G}) = \min_{a \in \{1,2,\ldots,A\}} \|\mathbf{s}_b - \mathbf{g}_a\|. \tag{22}$$

- Mean absolute distance measures the average difference between the ROI boundaries of S and G:

$$\text{MAD} = \frac{1}{B} \sum_{b=1}^{B} d(\mathbf{s}_b, \mathcal{G}). \tag{23}$$

- Maximum absolute distance represents the maximum difference between the ROI boundaries of S and G:

$$\text{MaxD} = \max_{b \in \{1,2,\ldots,B\}} d(\mathbf{s}_b, \mathcal{G}). \tag{24}$$

- The Hausdorff distance [25] measures the extent between the set of vertices $\mathcal{S}$ and $\mathcal{G}$, which correspond to the boundaries of regions S and G, respectively. It is defined as:

$$\text{HD} = \max\{h(\mathcal{G}, \mathcal{S}), h(\mathcal{S}, \mathcal{G})\}, \tag{25}$$

  where $h(\mathcal{G}, \mathcal{S}) = \max_{\mathbf{g} \in \mathcal{G}}\{\min_{\mathbf{s} \in \mathcal{S}}\{d(\mathbf{g}, \mathbf{s})\}\}$, $h(\mathcal{S}, \mathcal{G}) = \max_{\mathbf{s} \in \mathcal{S}}\{\min_{\mathbf{g} \in \mathcal{G}}\{d(\mathbf{s}, \mathbf{g})\}\}$, and $d(\mathbf{g}, \mathbf{s})$ denotes the Euclidean distance.

In the case of missing detection by an automated segmentation method (i.e., no contour $\mathcal{S}$ is available), we set all the aforementioned distance-based metrics to the maximum value of the point-wise distance (i.e., the diagonal of the CT image).

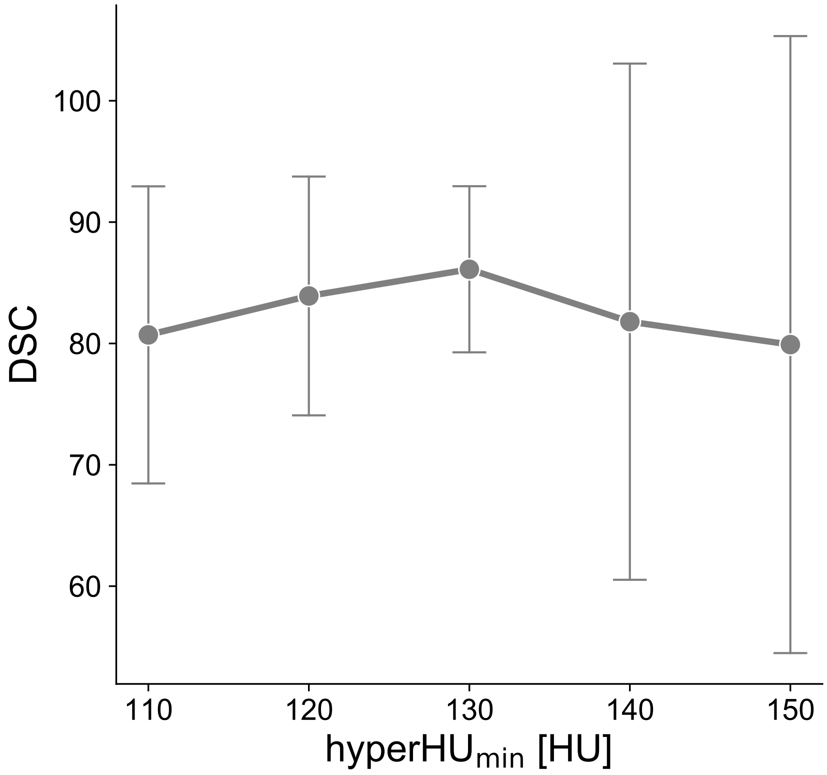

Figure S1: Comparison of the DSC achieved by the proposed method (employing the classic FCM version) on the calibration set for hyper-dense component segmentation considering $\mathsf{hyperHU}_{\text{min}} \in \{110, 120, 130, 140, 150\}$. The calibration set was composed of 70 CT images. The points and the error bars represent the average and standard deviation of the DSC values, respectively.

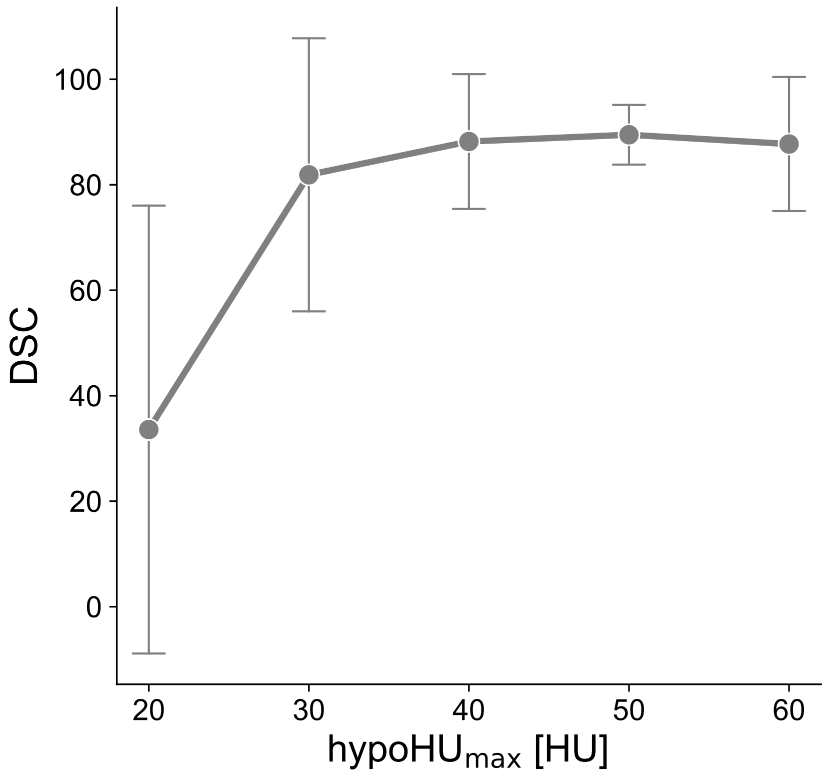

Figure S2: Comparison of the DSC achieved by the proposed method (employing the classic FCM version) on the calibration set for hypo-dense component segmentation considering $\mathsf{hypoHU}_{\mathrm{max}} \in \{20, 30, 40, 50, 60\}$, after setting $\mathsf{hyperHU}_{\mathrm{min}} = 130$ HU. The calibration set was composed of 120 CT images. The points and the error bars represent the average and standard deviation of the DSC values, respectively.

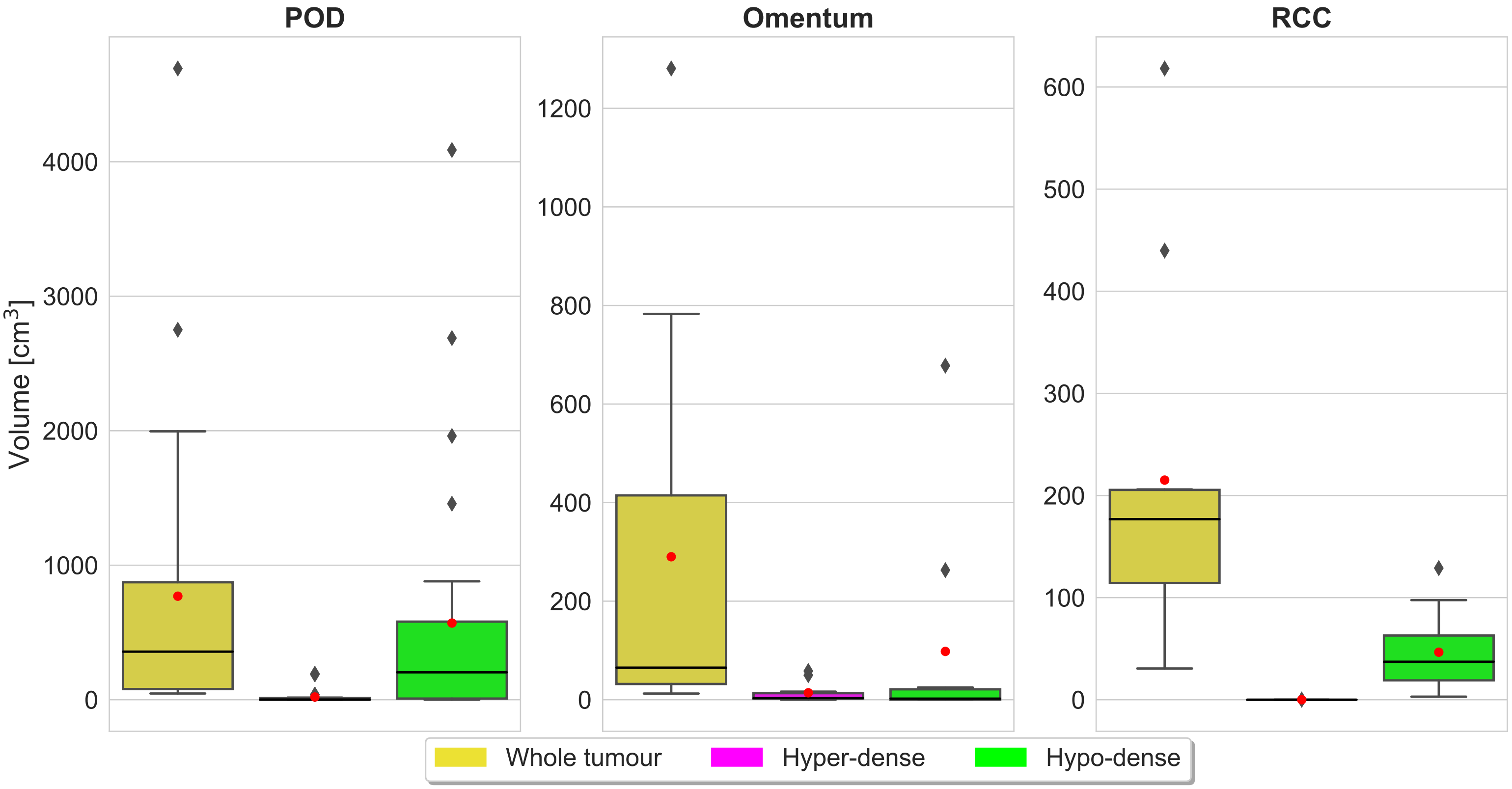

Figure S3: Volume measurements for the whole tumour, hyper-dense and hypo-dense components for the three tumour lesion locations (POD, omental, and RCC lesions) used for tissue-specific sub-segmentation. The black line and the red circular marker denote the median and the mean values, respectively. The diamonds represent the outliers.

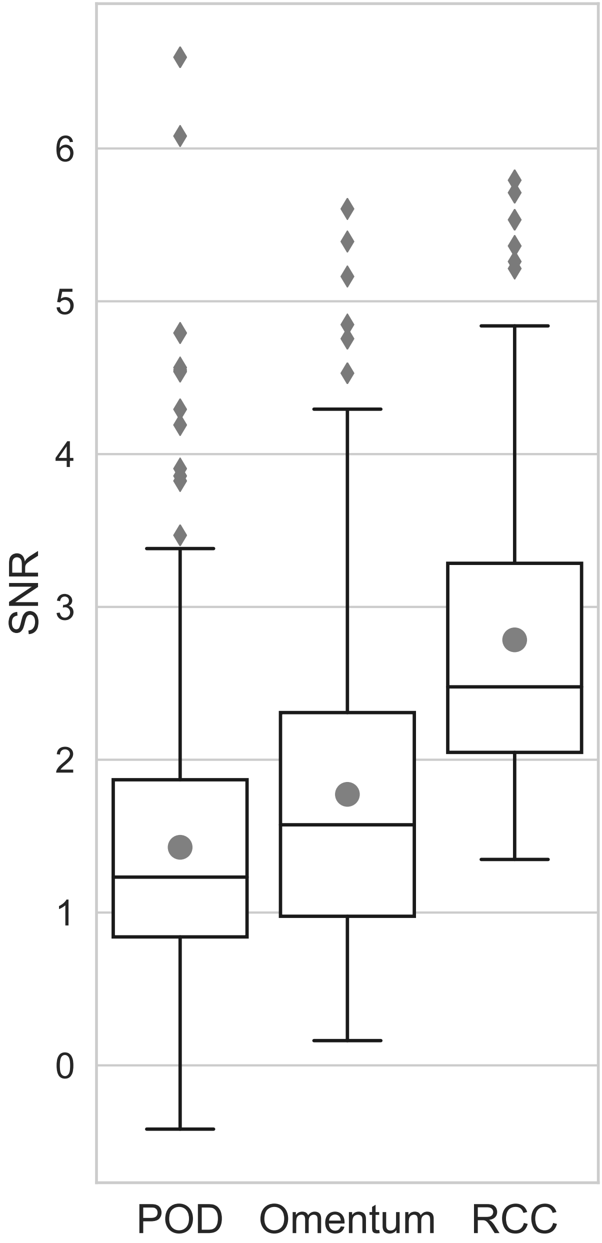

Figure S4: Signal-to-Noise-Ratio (SNR) values obtained on the three tumour lesion locations (POD, omental, and RCC lesions) used for tissue-specific sub-segmentation. The black line and the grey circular marker denote the median and the mean values, respectively. The diamonds represent the outliers.

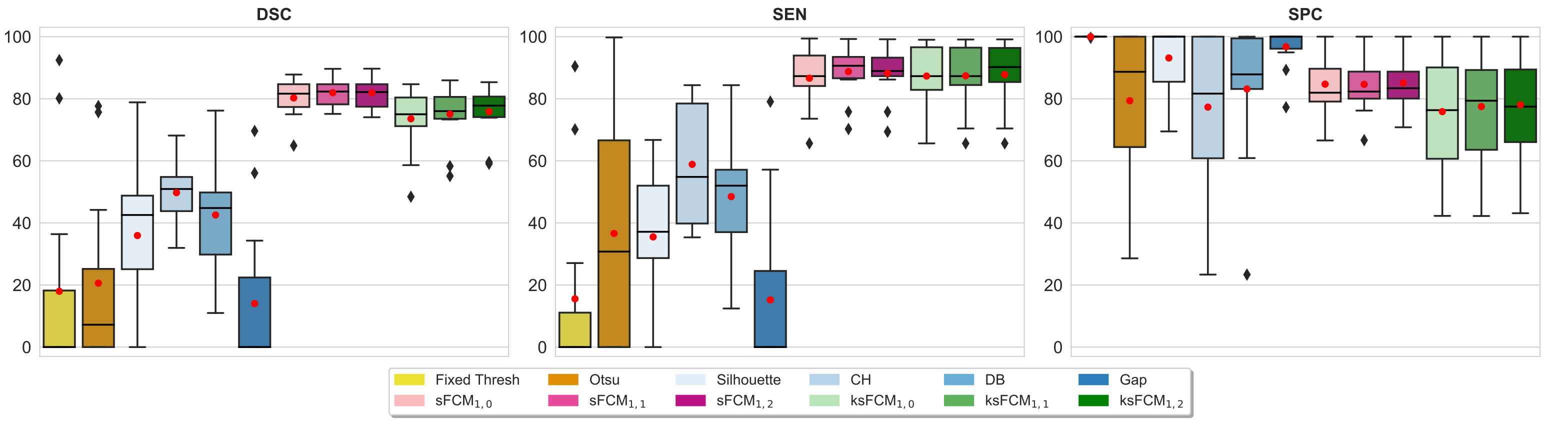

Figure S5: Spatial overlap-based segmentation metrics for the hyper-dense components of the POD lesions included in the ovarian cancer CT datasets.

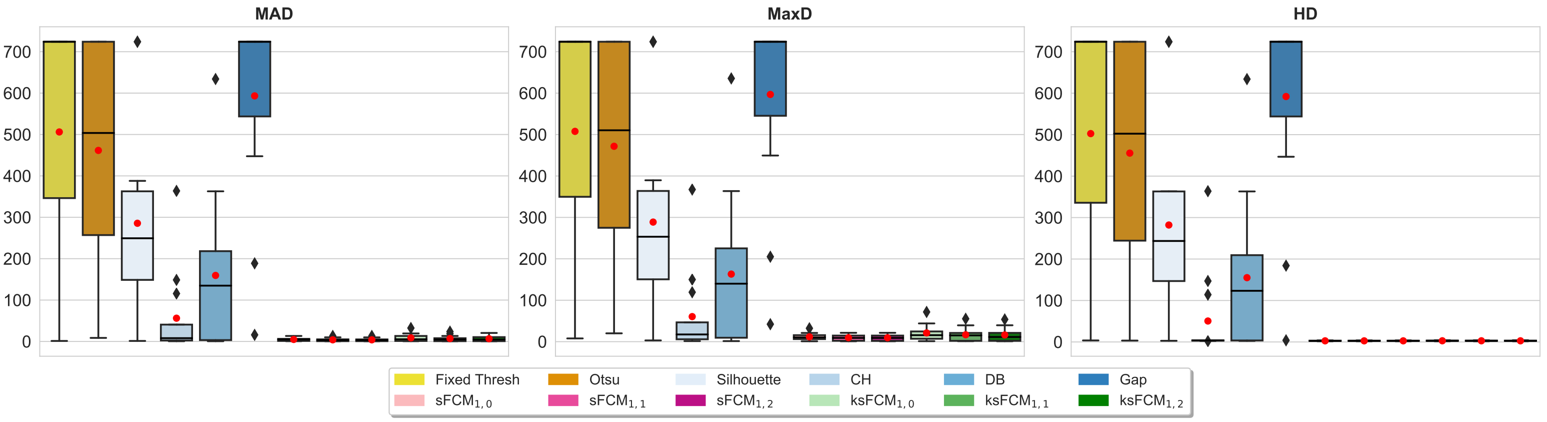

Figure S6: Spatial distance-based segmentation metrics for the hyper-dense components of the POD lesions included in the ovarian cancer CT datasets.

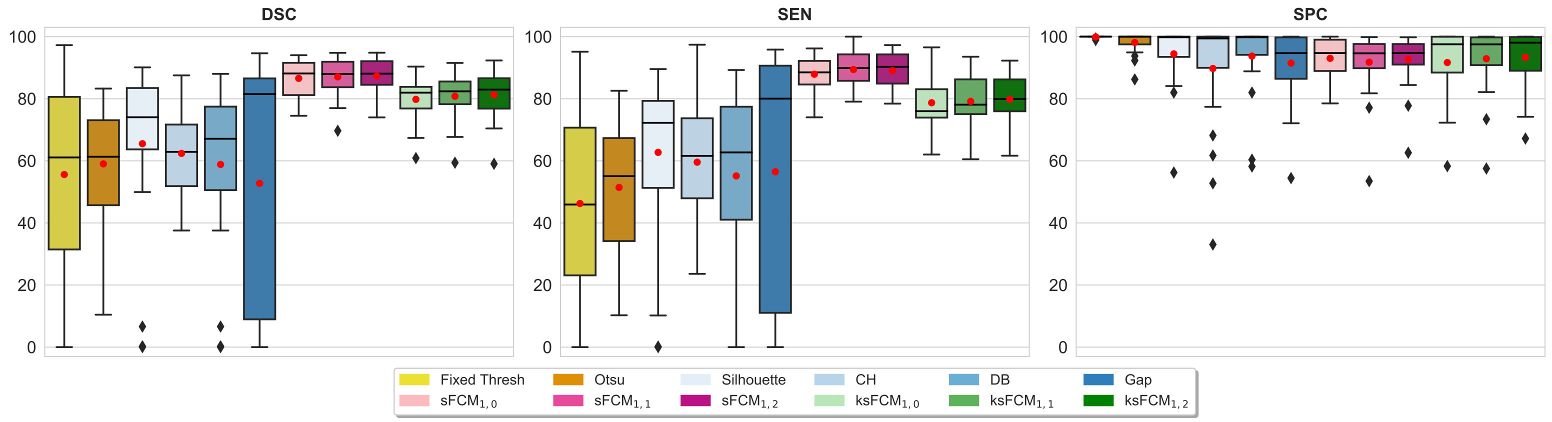

Figure S7: Spatial overlap-based segmentation metrics for the hypo-dense components of the POD lesions included in the ovarian cancer CT datasets.

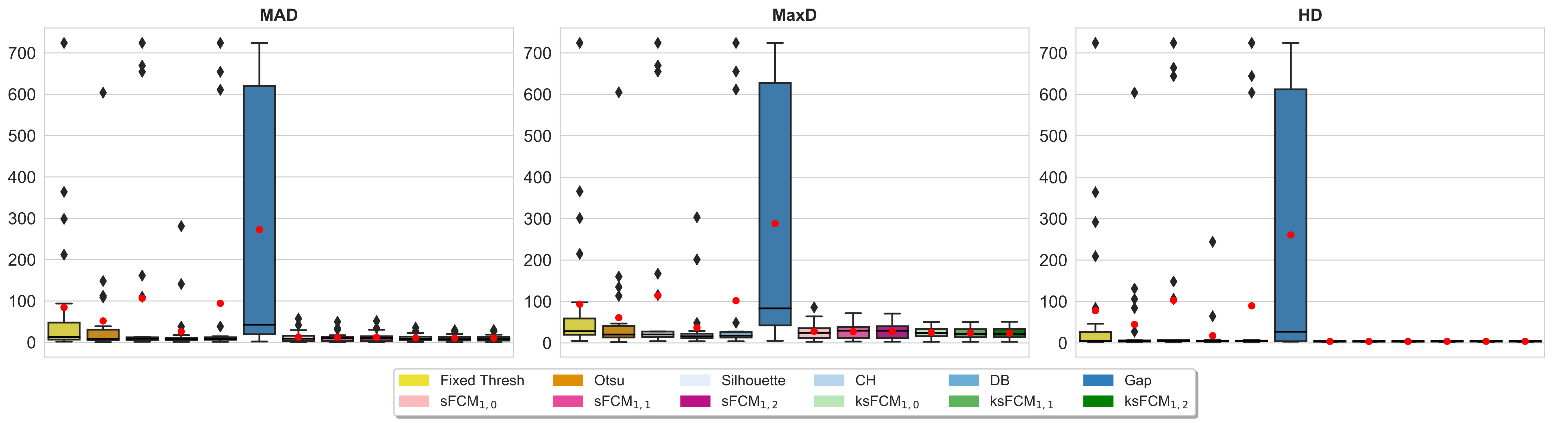

Figure S8: Spatial distance-based segmentation metrics for the hyper-dense components of the POD lesions included in the ovarian cancer CT datasets.

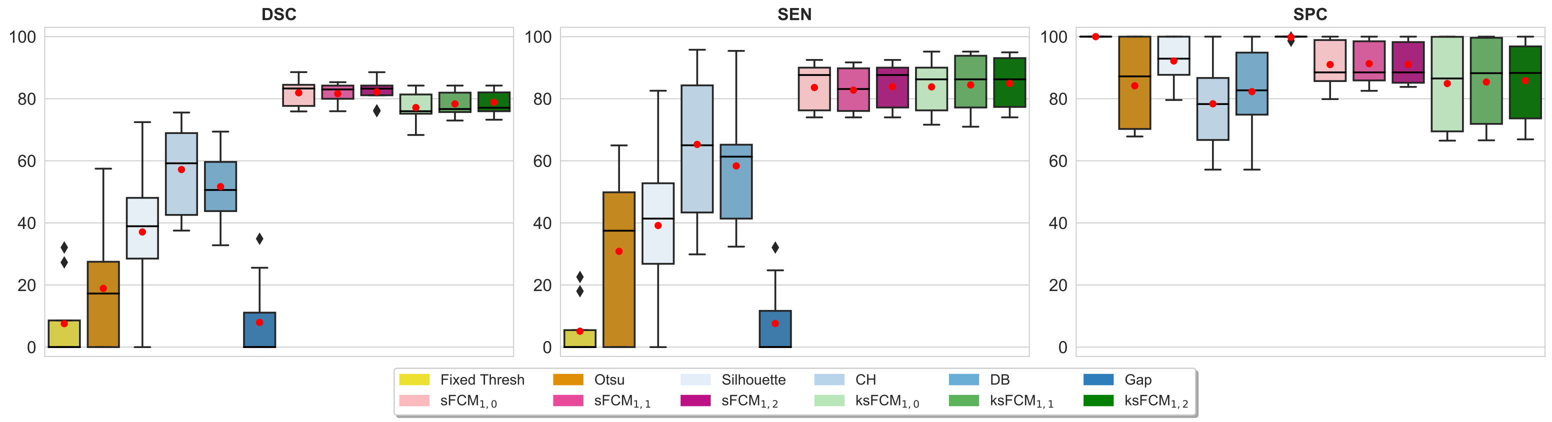

Figure S9: Spatial overlap-based segmentation metrics for the hyper-dense components of the omental lesions included in the ovarian cancer CT datasets.

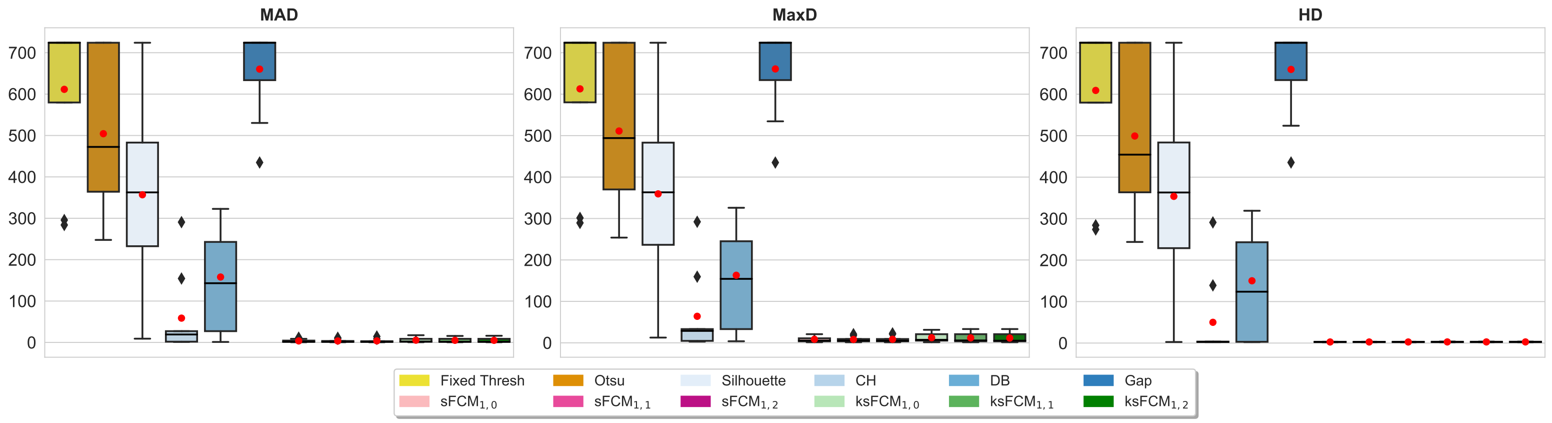

Figure S10: Spatial distance-based segmentation metrics for the hyper-dense components of the omental lesions included in the ovarian cancer CT datasets.

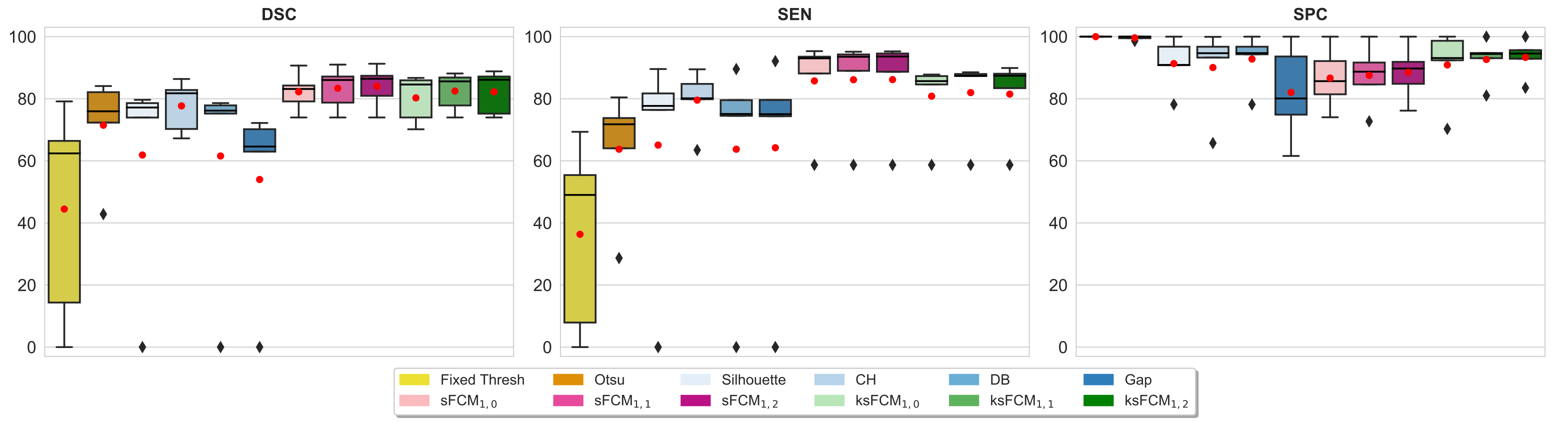

Figure S11: Spatial overlap-based segmentation metrics for the hypo-dense components of the omental lesions included in the ovarian cancer CT datasets.

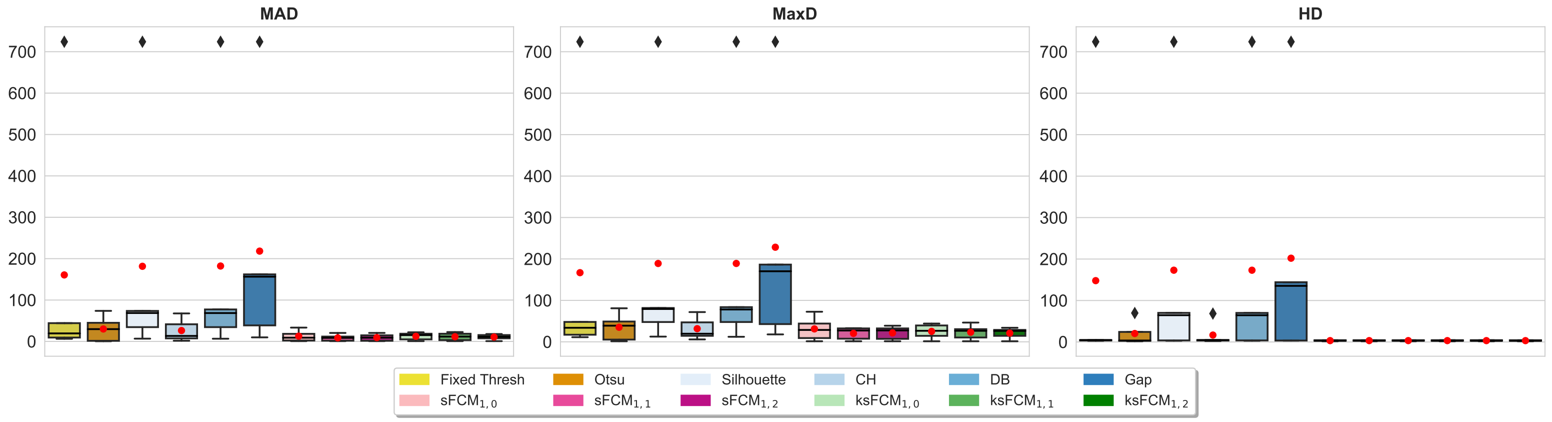

Figure S12: Spatial distance-based segmentation metrics for the hyper-dense components of the omental lesions included in the ovarian cancer CT datasets.

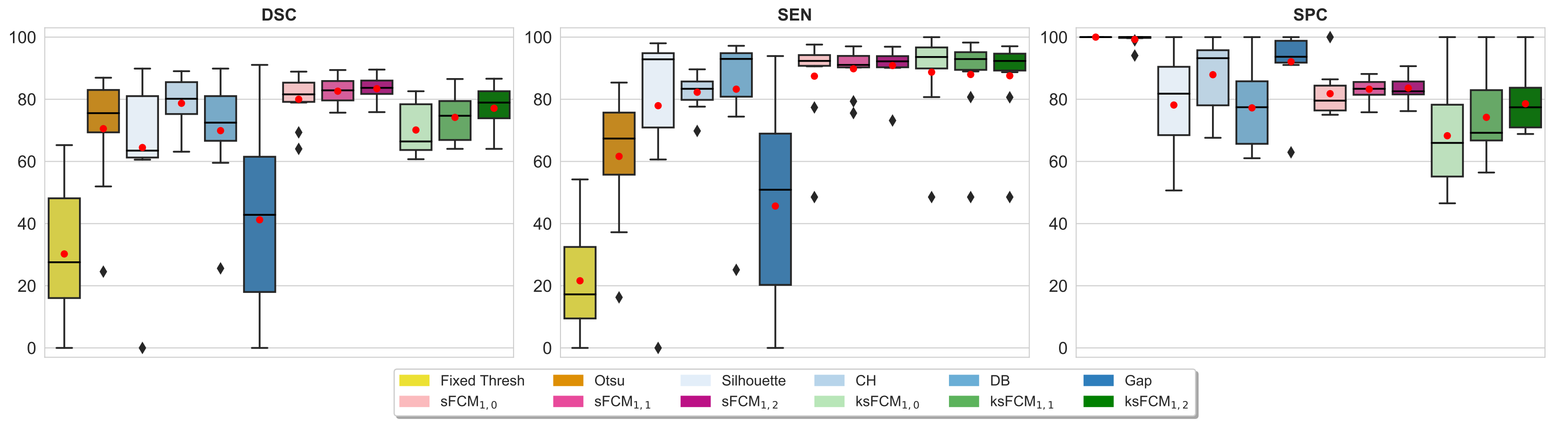

Figure S13: Spatial overlap-based segmentation metrics for the hypo-dense components of the RCC lesions included in the kidney cancer CT datasets.

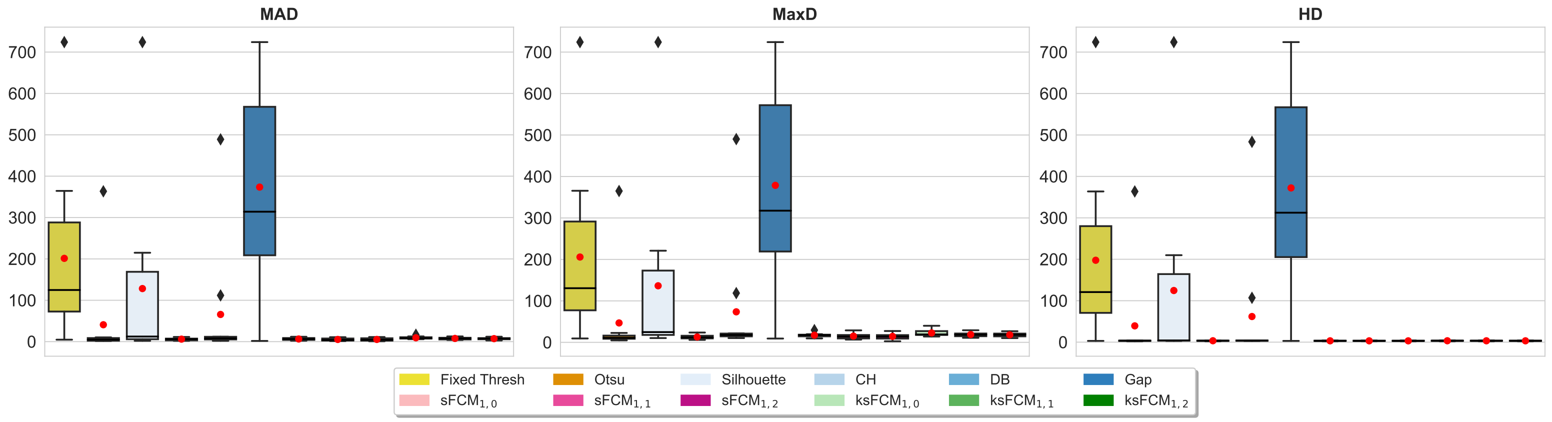

Figure S14: Spatial distance-based segmentation metrics for the hyper-dense components of the RCC lesions included in the kidney cancer CT datasets.

References

[1] J. C. Bezdek, Objective function clustering, in: Pattern Recognition with Fuzzy Objective Function Algorithms, 1st Edition, Springer-Verlag New York, Inc., Secaucus, NJ, USA, 1981, pp. 43–93. doi:10.1007/978-1-4757-0450-1.

[2] J. C. Bezdek, R. Ehrlich, W. Full, FCM: the fuzzy c-means clustering algorithm, Comput. Geosci. 10 (2-3) (1984) 191–203. doi:10.1016/0098-3004(84)90020-7.

[3] Y.-L. Li, Y. Shen, An automatic fuzzy c-means algorithm for image segmentation, Soft Comput. 14 (2) (2010) 123–128. doi:10.1007/s00500-009-0442-0.

[4] B. N. Li, C. K. Chui, S. Chang, S. H. Ong, Integrating spatial fuzzy clustering with level set methods for automated medical image segmentation, Comput. Biol. Med. 41 (1) (2011) 1–10. doi:10.1016/j.compbiomed.2010.10.007.

[5] S. Lloyd, Least squares quantization in PCM, IEEE Trans. Inform. Theory 28 (2) (1982) 129–137. doi:10.1109/TIT.1982.1056489.

[6] T. Kanungo, D. M. Mount, N. S. Netanyahu, C. D. Piatko, R. Silverman, A. Y. Wu, An efficient k-means clustering algorithm: analysis and implementation, IEEE Trans. Pattern Anal. Mach. Intell. 24 (7) (2002) 881–892. doi:10.1109/TPAMI.2002.1017616.

[7] L. A. Zadeh, Fuzzy sets, Inf. Control 8 (3) (1965) 338–353. doi:10.1016/S0019-9958(65)90241-X.

[8] J. K. Udupa, S. Samarasekera, Fuzzy connectedness and object definition: theory, algorithms, and applications in image segmentation, Graph. Models Image Process. 58 (3) (1996) 246–261. doi:10.1006/gmip.1996.0021.

[9] D.-Q. Zhang, S.-C. Chen, A novel kernelized fuzzy c-means algorithm with application in medical image segmentation, Artif. Intell. Med. 32 (1) (2004) 37–50. doi:10.1016/j.artmed.2004.01.012.

[10] H.-C. Huang, Y.-Y. Chuang, C.-S. Chen, Multiple kernel fuzzy clustering, IEEE Trans. Fuzzy Syst. 20 (1) (2012) 120–134. doi:10.1109/TFUZZ.2011.2170175.

[11] D. L. Pham, J. L. Prince, Adaptive fuzzy segmentation of magnetic resonance images, IEEE Trans. Med. Imaging 18 (9) (1999) 737–752. doi:10.1109/42.802752.

[12] N. R. Pal, J. C. Bezdek, On cluster validity for the fuzzy c-means model, IEEE Trans. Fuzzy syst. 3 (3) (1995) 370–379. doi:10.1109/91.413225.

[13] L. Caponetti, G. Castellano, V. Corsini, MR brain image segmentation: a framework to compare different clustering techniques, Information 8 (4) (2017) 138. doi:10.3390/info8040138.

[14] D. L. Pham, Spatial models for fuzzy clustering, Comput. Vis. Image Underst. 84 (2) (2001) 285–297. doi:10.1006/cviu.2001.0951.

[15] C. J. Daniels, F. A. Gallagher, Unsupervised segmentation of 5D hyperpolarized Carbon-13 MRI data using a fuzzy Markov random

field model, IEEE Trans. Med. Imaging 37 (4) (2017) 840–850. doi:10.1109/TMI.2017.2737232.

[16] M. Gong, Y. Liang, J. Shi, W. Ma, J. Ma, Fuzzy c-means clustering with local information and kernel metric for image segmentation, IEEE Trans. Image Process. 22 (2) (2012) 573–584. doi:10.1109/TIP.2012.2219547.

[17] S. Krinidis, V. Chatzis, A robust fuzzy local information c-means clustering algorithm, IEEE Trans. Image Process, 19 (5) (2010) 1328–1337. doi:10.1109/TIP.2010.2040763.

[18] K.-S. Chuang, H.-L. Tzeng, S. Chen, J. Wu, T.-J. Chen, Fuzzy c-means clustering with spatial information for image segmentation, Comput. Med. Imaging Graph. 30 (1) (2006) 9–15. doi:10.1016/j.compmedimag.2005.10.001.

[19] B. Schölkopf, The kernel trick for distances, in: Proc. Advances in Neural Information Processing Systems (NIPS), 2001, pp. 301–307.

[20] S. Chen, D. Zhang, Robust image segmentation using FCM with spatial constraints based on new kernel-induced distance measure, IEEE Trans. on Systems, Man, and Cybernetics, Part B (Cybernetics) 34 (4) (2004) 1907–1916. doi:10.1109/TSMCB.2004.831165.

[21] A. Taha, A. Hanbury, Metrics for evaluating 3D medical image segmentation: analysis, selection, and tool, BMC Med. Imaging 15 (1) (2015) 29. doi:10.1186/s12880-015-0068-x.

[22] A. Fenster, B. Chiu, Evaluation of segmentation algorithms for medical

imaging, in: Proc. Annual International Conference of the IEEE Engineering in Medicine and Biology Society, IEEE, 2005, pp. 7186–7189. doi:10.1109/IEMBS.2005.1616166.

[23] Y. Zhang, A review of recent evaluation methods for image segmentation, in: Proc. IEEE International Symposium on Signal Processing and its Applications (ISSPA), Vol. 1, IEEE, 2001, pp. 148–151. doi:10.1109/ISSPA.2001.949797.

[24] K. Zou, S. Warfield, A. Bharatha, C. Tempany, M. Kaus, S. Haker, et al., Statistical validation of image segmentation quality based on a spatial overlap index, Acad. Radiol. 11 (2) (2004) 178–189. doi:10.1016/S1076-6332(03)00671-8.

[25] R. Cárdenes, R. de Luis-García, M. Bach-Cuadra, A multidimensional segmentation evaluation for medical image data, Comput. Methods Programs Biomed. 96 (2) (2009) 108–124. doi:10.1016/j.cmpb.2009.04.009.
